# Gut Microbiota Differences According to Ultra-Processed Food Consumption in a Spanish Population

**DOI:** 10.3390/nu13082710

**Published:** 2021-08-06

**Authors:** Amanda Cuevas-Sierra, Fermín I. Milagro, Paula Aranaz, Jose Alfredo Martínez, José I. Riezu-Boj

**Affiliations:** 1 Department of Nutrition, Food Sciences and Physiology, University of Navarra, 31008 Pamplona, Spain; acuevas.1@alumni.unav.es (A.C.-S.); jalfmtz@unav.es (J.A.M.); jiriezu@unav.es (J.I.R.-B.); 2 Center for Nutrition Research, University of Navarra, 31008 Pamplona, Spain; paranaz@unav.es; 3 Centro de Investigación Biomédica en Red de la Fisiopatología de la Obesidad y Nutrición (CIBERobn), Institute of Health Carlos III, 28029 Madrid, Spain; 4 Navarra Institute for Health Research (IdiSNA), 31008 Pamplona, Spain

**Keywords:** microbiota, ultra-processed food, sex-differences, *Bifidobacterium*, Bacteroidetes

## Abstract

Ultra-processed foods (UPFs) consumption could affect gut microbiota diversity and profile. We aimed to evaluate the effects of UPFs on microbiota, considering the role of sex. The consumption of UPFs (using NOVA criteria) was assessed with a validated 137-item food-frequency questionnaire. Participants (*n* = 359) were classified into less than three servings per day (*n* = 96) of UPFs and more than five (*n* = 90). Women and men were subclassified following the same criteria. 16S rRNA sequencing was performed from DNA fecal samples, and differences in microbiota were analyzed using EdgeR. The relationship between UPFs and bacteria was assessed by Spearman correlation and comparison of tertiles of consumption. Women who consumed more than five servings/day of UPFs presented an increase in *Acidaminococcus, Butyrivibrio, Gemmiger, Shigella, Anaerofilum*, *Parabacteroides, Bifidobacterium,* Enterobacteriales, Bifidobacteriales and Actinobacteria and a decrease in *Melainabacter* and *Lachnospira*. *Bifidobacterium*, Bifidobacteriales and Actinobacteria was positively associated with pizza and Actinobacteria with industrially processed dairy in women. Men who consumed more than five servings/day presented an increase of *Granulicatella, Blautia,* Carnobacteriaceae, Bacteroidaceae, Peptostreptococcaceae, Bacteroidia and Bacteroidetes and a decrease of *Anaerostipes* and Clostridiaceae. Bacteroidia and Bacteroidetes correlated positively with industrially processed meat. This study suggests that UPFs may affect microbiota composition differently in women and men.

## 1. Introduction

Gut microbiota may be affected by different factors such as stress, aging, type of birth, antibiotics or probiotics use, physical activity, gastrointestinal diseases, etc. [1]. However, food seems to be a crucial variable that can modulate the gut microbiota composition, and subsequently influence nutrient absorption, regulation of nutrient harvest and different metabolic outcomes [2]. Diet provides the energy intake and nutrients required for humans, but also the sustenance for gut bacteria. Thus, the type of diet will consequently impact on the selective growth of bacterial taxa in the human gut. Regarding dietary patterns, the beneficial impact of a healthy diet rich in fruit, vegetables and fresh products on the gut has been largely reported [3]. However, the consumption of fresh-food is decreasing whereas the intake of ultra-processed foods (UPFs) is increasing worldwide [4]. Over the last few decades, traditional and fresh food are being replaced by packaged UPFs in many countries, especially in high-income countries [5]. UPFs are formulations ready for consumption, made from refined food substances (such as glucose syrup, modified starch, maltodextrins, hydrogenated oils, protein/fiber isolates or cosmetic additives), with a careful combination of simple sugars, salt, fat, and various additives. These foods, which include sugar-sweetened beverages (SSB), snacks, industrially processed pastries, meat and dairy products, and “fast foods”, among others, are not recommended for prolonged consumption. Their low nutritional quality, high energy density [6], and hyper-palatable attributes also promote overconsumption [7]. Some ecological studies have shown that the increased consumption of UPFs has coincided with an increasing prevalence of obesity. In fact, several studies have found associations between UPFs and health problems such as hypertension [8], obesity [9], metabolic syndrome [10], depression [11] and type 2 diabetes [12]. These pathologies are also connected with oxidative stress and inflammation, which could modify gut microbiota configuration, richness and diversity [13]. Diet is partly responsible for the homeostasis of the microbiota, as short-term modifications in the dietary pattern carry variations in diversity and composition.

Others factors can also affect gut microbiota composition, including genetic background [14], physical exercise [15], aging [16] or sex [17]. In this context, several studies have demonstrated that sex must be taken into account since gut microbiota composition differs between men and women. Hormone concentration, adiposity, fat distribution [18,19,20,21,22], and different eating behavior and habits [23] could explain these differences. However, quite often this factor has been ignored. Although some studies have taken into account the differences between sex in the consumption of UPFs [24], changes in the microbiota produced by UPFs according to sex should be further studied. In this study we hypothesized that UPFs consumption can affect gut microbiota, but in a different manner depending on sex. Thus, the aim of this work was to assess the effects of UPFs consumption on human gut microbiota composition in normal weight, overweight and obese Spanish populations, taking into account the role of sex.

## 2. Materials and Methods

### 2.1. Subjects

This study includes baseline data from 296 Caucasian adults with body mass index (BMI) from 25 to 36 kg/m^2^ from the Obekit trial (NCT02737267). Also included were 63 subjects with normal weight (BMI from 18 to 24.9 kg/m^2^). The characteristics of this research project, the study design, and the exclusion and inclusion criteria have been previously detailed [25,26]. A written informed consent was signed and the study protocol was approved by the Research Ethics Committee of the University of Navarra (ref. 132/2015). Throughout the project, the Ethical principles of the Helsinki Declaration were rigorously followed. [27]. A total of 359 participants were finally included in this investigation. Participants who did not provide fecal samples in a correct manner or consumed antibiotics before collecting the samples (*n* = 12) or did not fulfill the food frequency questionnaire (*n* = 1) were discarded.

### 2.2. Assessment of Ultra-Processed Food Consumption

Habitual dietary intake at baseline was collected with a food frequency questionnaire (FFQ) validated in Spain that included 137 food items with corresponding portion sizes as described elsewhere [28]. Participants provided information about the number of times they had consumed each food item during the last year. The answer corresponded to nine categories for consumption frequencies (from never/almost never to >6 servings per day). Total energy (kcal) and macronutrient intakes (%) were determined using an ad hoc computer program specifically developed for this purpose, by calculating this as the sum of frequency of consumption multiplied by nutrient composition of a specified portion size from valid Spanish food composition tables [29]. All items in FFQ were classified according to the NOVA system, a food classification based on the nature, extent, and purpose of industrial processing. Classification in the NOVA system corresponds to: 1. ‘unprocessed/minimally processed foods’ belonging to animals or plants (fruits, vegetables, eggs, milk, meat, etc.) which are fresh or very low-processed in ways that do not add substances such as salt, sugar, oils, fats, or additives; 2. substances extracted from unprocessed foods (from group 1) which are processed using culinary ingredients (salt, oil, sugar, etc.); 3. processed foods made by adding processed culinary ingredients to minimally processed food (such as fruit in syrup or cheese); and 4. UPFs, which are defined as industrial formulations of food-derived substances (oils, fats, sugars, modified starch, protein isolates) that include little or no whole food and often with flavorings, colorings, emulsifiers, and other cosmetic additives (e.g., sweet and savory packaged snacks, chocolate snacks and candies, mass-produced packaged buns, cookies, industrially produced cakes, breakfast “cereals”, ready-to-heat or pre-fried products, instant soups, industrial pizza, sausages or hamburgers). The processes for manufacturing include sophisticated technology and techniques such as hydrogenation, hydrolysis, extrusion, molding, remodeling, pre-frying or adding several additives to increase palatability. The techniques of manufacturing UPFs includes the addition of flavor enhancers, emulsifiers, thickeners, and anti-foaming, bulking, gelling, carbonating, foaming, glazing agents and additives that help to prolong product duration [30,31,32]. The sum of total consumed UPFs was calculated using the frequency of UPF items consumption per person (servings per day). Supplementary Table S1 shows the UPF products included in the FFQ, previously used in other publications [9,33].

### 2.3. Fecal Sample Collection and Metagenomic Data

Fecal samples were self-collected by the volunteers using OMNIgene.GUT kits from DNA Genotek (Ottawa, ON, Canada) and following the standard guidelines from the supplier. Samples were aliquoted and stored at −80 °C. The isolation of DNA was achieved with the QIAamp^®^ DNA kit (Qiagen, Hilden, Germany) following the manufacturer’s protocol. Bacterial DNA sequencing was carried out in the Autonomous University of Barcelona, (Servei de Genòmica i Bioinformàtica) in Spain. The Illumina 16S protocol based on the amplification of the V3-V4 variable regions of the 16S rRNA gene was followed for sequencing. This process consists of two PCRs. In the first one, 12.5 ng of genomic DNA and the 16S Amplicon PCR Forward and 16S Amplicon PCR Reverse primers were used (from Nextera^®^ XT DNA Index Kit FC-131-1002 Illumina; San Diego, CA, USA). The protocol in this first PCR was 95 °C during 3 min and 25 cycles of: 95 °C for 30 s, 55 °C for 30 s, 72 °C for 30 s, then, finally, 72 °C for 5 min and hold at 4 °C. The protocol for the second PCR was 95 °C for 3 min, 8 cycles of: 95 °C for 30 s, 55 °C for 30 s, 72 °C for 30 s, then, finally, 72 °C for 5 min and hold at 4 °C. The PCR quality was assessed in a Labchip Bioanalyzer (Agilent Technologies Inc, Santa Clara, CA, USA). Once the sequencing of all the samples had been achieved, up to 40 samples were multiplexed in each run of 2 × 300 cycles. Equimolar concentrations of each samples were mixed and the pool diluted up to 20 pM. A total of 3 runs were performed on the MiSeq sequencer using the MiSeq^®^ Reagent Kit v3 (San Diego, CA, USA) (600 cycle) MS-102-3003. During the process, negative controls were included. In order to avoid batch effect, samples were randomized by sex, age and BMI status. Adapters and barcodes were removed following the Illumina guidelines. Acceptable quality readings were considered when obtained at a sequence depth of 40,000 readings as minimum. The 16S rRNA sequences were trimmed and filtered following quality criteria of the processing pipeline LotuS (release 1.58) for MiSeq sequencer [34]. This pipeline includes UPARSE de novo sequence clustering for the identification of Operational Taxonomic Units (OTUs) and their abundance matrix generation [35,36] by similarities in DNA sequence, with a sequence similarity threshold of 97%. [37]. Taxonomy was assigned using HITdb database for human intestinal 16S rRNA sequences [38]. The abundance matrices were filtered and normalized in R/Bioconductor from OTU to phylum [39]. Sequencing data of this study can be found in the NCBI SRA repository (accession number PRJNA623853).

### 2.4. Anthropometric Measurements

Anthropometric measurements were collected by trained nutritionists using conventional validated methods. BMI was calculated and classified according to the World Health Organization [40]. Fat distribution was assessed using Dual-energy X-ray absorptiometry, following the company instructions (DEXA, Lunar Prodigy, software version 6.0, Madison, WI, USA). Systolic and diastolic blood pressures were measured using conventional methods (with a sphygmomanometer), following the criteria of the World Health Organization and the International Society of Hypertension [41]. Trained nutritionist collected information including lifestyle-related characteristics, smoking status and clinical history. Adherence to the Mediterranean dietary pattern was assessed using a validated score [42]. A validated 17-items questionnaire was used for the assessment of physical activity of the participants (expressed as metabolic equivalents units, METS), as detailed elsewhere [43].

### 2.5. Biochemical Measurements

Blood samples were collected after 12 h of overnight fasting. Glucose, total cholesterol, high-density lipoprotein cholesterol (HDL-c), triglycerides, alanine-aminotransferase (ALT), and aspartate-aminotransferase (AST) were tested in an automatized analyzer Pentra C200 by using suitable kits provided by the company (HORIBA Medical, Madrid, Spain). Low-density lipoprotein cholesterol (LDL-c) was calculated using the Friedewald equation (LDL-c = Total cholesterol (mg/dL) − HDL-c (mg/dL) − triglycerides (mg/dL)/5) [44]. Homeostatic model assessment for insulin resistance (HOMA-IR) was calculated using fasting insulin and glucose concentrations [45]. Insulin, adiponectin, leptin, tumor necrosis factor alpha (TNF-α) and C-reactive protein (CRP) were measured with specific enzyme-linked immunosorbent assays and read with an automated analyzer system (Triturus, Grifols, Barcelona, Spain). The following kits were used: insulin (Mercodia, Uppsala, Sweden), TNF-α (R&D Systems, Minneapolis, MN, USA), CRP (Demeditec, Kiel, Germany), adiponectin (BioVendor, Brno, Czech Republic), leptin (Mercodia, Uppsala, Sweden), following the instructions from the suppliers.

### 2.6. Statistical Analysis

The total servings per day of UPFs was adjusted for BMI, age and energy intake at baseline through the residual methods. Then, the entire population (*n* = 359) was categorized into two groups according to the consumption of UPFs: less than 3 UPFs servings per day (*n* = 96) and more than 5 UPFs servings per day (*n* = 90). A total of 186 subjects were included for the comparative analysis. For the analysis separated by sex, women subjects (*n* = 251) were classified into women who consumed less than 3 servings/day (*n* = 57) of UPFs and more than 5 (*n* = 66). The same classification was applied for male participants (*n* = 108), men who consumed less than 3 servings/day (*n* = 39) of UPFs and more than 5 (*n* = 24). Thus, subjects who consumed between 3 and 5 servings per day of UPFs were excluded (*n* = 173). In this way, a “high” consumption group (<3 servings/day) and a “low” consumption group (>5 servings/day) were obtained. Descriptive statistics were used to analyze baseline characteristics among participants using Stata 16 (StataCorp LLC, College Station, TX, USA). Variables were expressed as means ± standard error of the mean for quantitative variables and as numbers of cases for qualitative variables. The data distribution was screened with the Shapiro–Wilk test. Biochemical and anthropometrical value differences at baseline between groups were assessed by t-test and Mann–Whitney test depending on the distribution of data, and quantitative value variables were assessed by Chi-squared. Comparative analysis of microbiota according to UPFs consumption (adjusted by BMI, age and energy intake in order to avoid potential confounders) was performed using MicrobiomeAnalyst [46] from phylum to genus. Microbiota data were filtered removing features with less than 4 counts and less than 20% of prevalence, as a minimum. From a total of 4734 features, 3884 were removed due to low abundance based on prevalence and another 33 features were removed based on inter-quantile range. Richness (number of species in our population) was calculated with the number based on OTU counts and alpha diversity (mean of different species within subject) indexes Chao1 and Shannon, analyzed using a paired non-parametric test. Beta diversity (mean of different species between subjects) was calculated using Bray Curtis index and PERMANOVA test. Statistical differences in microbiota abundances between groups of adjusted UPFs consumption were tested by EdgeR (Empirical analysis for Digital Gene Expression in R) previously normalized using trimmed mean of values normalization (TMM) and false discovery rate for correction (considered statistically significant when FDR < 0.05). The association between microbiota and categories of ultra-processed food was evaluated by Spearman correlation using Stata 16 separately in women and men (taking into account the whole population without separating by UPF consumption categories) and corrected by FDR (considered significant when FDR > 0.05). Correlation graphs were performed using GraphPad Prism version 6.0 (GraphPad Software, San Diego, CA, USA). Differences in bacterial abundance according to adjusted UPFs consumption were assessed using tertiles of consumption for women (89 subjects in each tertile) and for men (31 subjects in each tertile) and comparing tertiles 1 and 3 by Mann-Whitney test using Stata 16.

## 3. Results

The consumption of adjusted UPFs (by BMI, age and energy intake) per day in our study population ranged from 0 to 18 serv/d and the mean of consumption was 4.0 ± 1.8. In women, adjusted UPFs consumption ranged from 0 to 10 and in men from 0 to 17. The mean consumption was 4.1 ± 1.7 for women and 3.8 ± 2.2 for men.

### 3.1. Characteristics of the Population

Table 1 shows the comparison of baseline characteristics according to the consumption of less than 3 servings per day and more than 5. Smoking, alcohol and physical exercise were not different between groups. The prevalence of anxiety and depression were significantly higher in participants who consumed more than 5 servings/day compared with those who consumed less than 3.

In the whole population, participants who consumed more than 5 servings/day showed a significantly higher energy intake, BMI, body weight, waist circumference, hip circumference, fat mass, and triglyceride levels. HDL-c levels were significantly lower in subjects who consumed more than 5 serv/d. Subjects with higher consumption of UPFs also presented an older age, a lower adherence to Mediterranean diet and a higher percentage of energy from UPFs (Table 1). In the female population (Table 1), the participants who consumed more than 5 serv/day of UPF presented more cases of depression and anxiety, energy intake and higher weight and hip circumference. The percentage of energy from UPFs was also significantly higher in the women who consumed more than 5 serv/d of UPFs (Table 1). On the contrary, the adherence to the Mediterranean diet was significantly lower when the UPFs consumption was higher than 5. Considering the male population, those that consumed more than 5 serv/d presented a significantly higher energy intake, BMI, weight, triglycerides, ALT, and TNF levels. On the contrary, levels of HDL-c were lower in the men who consumed more UPFs. Men who consumed more than 5 serv/day of UPFs also showed a significantly lower adherence to the Mediterranean diet and a higher percentage of energy from UPFs (Table 1). PAD, PAS, visceral fat, blood glucose, insulin, HOMA index, total and LDL cholesterol, AST, adiponectin, leptin, and CPR showed no significant differences between groups of consumption (Table 1). Supplementary Table S2 shows characteristics of the participants excluded for the microbiota analyses (those subjects who consumed between 3 and 5 servings per day of UPFs).

### 3.2. Consumption of the Different Groups of Ultra-Processed Food

Table 2 shows the averages of consumption for each UPF group in the whole population, women, and men, depending on the consumption of less than 3 UPFs per day and more than 5 (adjusted by BMI, age and energy intake). Regarding the consumption of the adjusted UPF in the whole population, significant differences were found in every group of food (excepting cereals and alcohol), showing that the participants who consumed more than 5 servings/day of UPF ate more from almost all groups of UPFs (Table 2). Similarly, the women who consumed more than 5 adjusted UPF showed a significant increase of consumption in every group of UPF, excepting cereals, margarine, ready-to-eat food and alcohol (Table 2). The men who consumed more than 5 adjusted UPF showed a significant increase in industrially processed meat, fried food, ready-to-eat products, cookies, pastries and SSB. However, the consumption of industrially processed dairy products was significantly higher in the men who consumed less than 3 servings/day of UPF (Table 2).

The comparison between men and women who consumed less than 3 adjusted UPFs revealed significant differences in alcohol consumption, being higher in men. On the other hand, men and women who consumed more than 5 adjusted UPFs presented significant differences in industrially processed meat (more consumed by women), alcohol and SSB (more consumed by men). The consumption of industrially processed dairy, cereals, pizza, margarine, fried food, cookies, light products, ready products, mayonnaise and pastries was similar in both sexes.

### 3.3. Analysis of Gut Microbiota Diversity According to Adjusted UPFs Consumption

**Figure 1:**
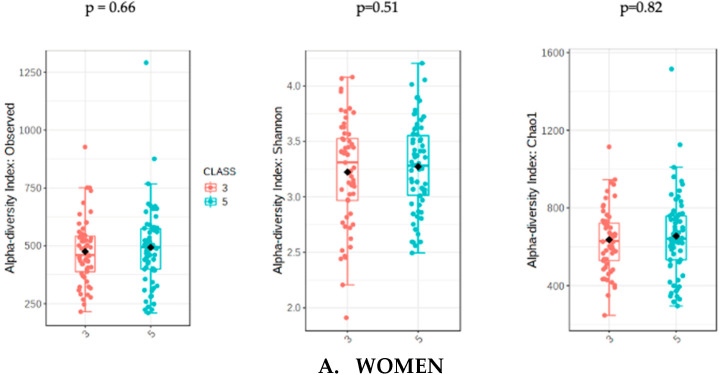

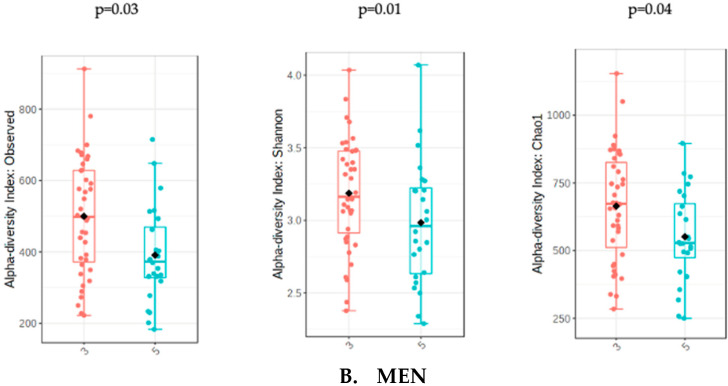
Richness measured as observed counts (left), Shannon diversity (middle) and Chao1 index (right), categorized according to the consumption of UPFs adjusted by BMI, age and energy intake for women (**A**) and men (**B**). Men who consumed more than 5 UPFs showed significantly lower richness and alpha diversity. Orange boxes represent women (**A**) and men (**B**) who consumed less than 3 serv/d of adjusted UPFs, and blue boxes represent the group that consumed more than 5 serv/d of adjusted UPFs.

The analysis of microbiota was evaluated with the sum of UPFs consumption (serv/d) adjusted by BMI, age and energy intake in order to avoid potential confounders. The analysis of gut microbiota richness between subjects who consumed less than 3 servings/day of UPFs and more than 5 showed no significant differences in the whole population (*p* = 0.31) and in women (*p* = 0.51). However, men who consumed more than 5 serv/d of UPFs showed a significant lower richness value compared to men who consumed less than 3 (*p* = 0.01). The analysis of alpha diversity in the whole population showed no significant differences when evaluated by Shannon (*p* = 0.61) and Chao1 (*p* = 0.38). Similarly, no significant differences in alpha diversity were found in the female subpopulation using Shannon (0.82) and Chao1 (*p* = 0.66). However, the men who consumed more than 5 serv/d of UPF presented a significantly lower alpha diversity when assessed by Shannon (*p* = 0.04) and Chao1 (*p* = 0.03) indexes (Figure 1).

Beta diversity presented no significant differences in any group.

### 3.4. Analysis of Gut Microbiota Composition According to UPF Consumption

Table 3 shows the comparison of the gut microbiota profile between the population who consumed less than 3 serv/d of adjusted UPF and more than 5 (Table 3) by using EdgeR. The participants who consumed more than 5 serv/d presented significantly higher abundance of *Gemmiger, Granulicatella, Parabacteroides, Shigella, Bifidobacterium, Anaerofilum, cc_115, Oxalobacter* and *Collinsella* genera. On the other hand, *Lachnospira* and *Roseburia* genera were underrepresented in the subjects who consumed more than 5 serv/d of adjusted UPFs. At family level, Carnobacteriaceae, Oxalobacteriaceae and Bifidobacteriaceae presented a significantly higher abundance in subjects who ingested more than 5 serv/d. At order level, Bifidobacteriales were overrepresented and Pasteurellales were underrepresented in subjects who consumed more than 5 serv/d. At class and phylum level, Actinobacteria class and Actinobacteria phylum were significantly overrepresented in the group who consumed more than 5 serv/d of UPFs (Table 3).

The analysis of the gut microbiota profile separated by sex showed distinctive results.

On the one hand, women (Table 4) who consumed more than 5 serv/d of UPFs presented a significant increase in *Acidaminococcus*, *Butyrivibrio*, *Gemmiger*, *Shigella*, *Anaerofilum*, *Parabacteroides* and *Bifidobacterium*. However, *Melainabacter* and *Lachnospira* were significantly less abundant in women who consumed more UPFs. No significant differences were found at family level, but at order level, Enterobacteriales and Bifidobacteriales showed an significant increase of abundance in women who ate more than 5 serv/d. Actinobacteria class was also more abundant in women who consumed more than 5 serv/d.

On the other hand, men (Table 5) who consumed more than 5 serv/d of adjusted UPF presented a significant increase of abundance in *Granulicatella* and *Blautia* genera, but a decrease in *Anaerostipes.* At family level, Carnobacteriaceae, Bacteoroidaceae and Peptostreptococcaceae were significantly more abundant in men who ate more UPF. Bacteroidia and Bacteroidetes also showed a significant higher abundance in this group.

Supplementary Tables S3–S5 shows the set of non-significant bacteria found between groups of UPFs consumption in whole population, women and men, respectively.

### 3.5. Analysis of Associations between Bacterial Taxa and Groups of UPFs

**Figure 2:**
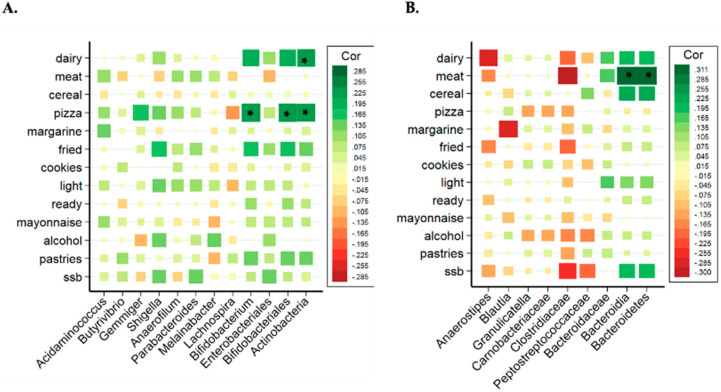
Correlation analysis of the significant bacterial taxa related to different types of UPFs in women (**A**) and in men (**B**). Spearman correlation coefficients and the corresponding *P* values were calculated based on comparisons of the relative abundance from genus to phylum and the consumption per day of UPF groups. * Adjusted *p* < 0.05. Correction for multiple comparisons used the false discovery rate (FDR; threshold of 0.05). Industrially processed dairy consumption includes cream, smoothies, milk drinks with or without flavors, flavored yogurts, custard, puddings and ice-creams; industrially processed meat consumption includes ham, smoked bacon, chorizo, mortadella, salami, sausage, hamburger, pate, spicy sausage, black pudding mortadella and meatballs; cookie consumption includes cookies and chocolate cookies; ready-to-eat food consumption includes instant soups and creams, instant pasta, croquettes and powdered soups and purees; pastries consumption: packaged buns, pre-prepared pies, prepared cakes, muffins, doughnuts, croissant or other business-type pastries, churros, chocolates and candies, nougat and marzipan.

Due to the distinctive results of the microbiota analysis, the relationship between bacterial taxa and the consumption of the different groups of ultra-processed products were studied separated by sex. Figure 2A,B show the correlation between groups of UPF with the significant bacteria found in all women and men (without categorizing by adjusted UPF consumption). In women, the consumption of industrially processed dairy products and pizza showed a positive and strong association with *Bifidobacterium*, Bifidobacteriales and Actinobacteria (class and phylum). However, the correlation matrix showed different results in men (Figure 2B), where Bacteroidia (class) and Bacteroidetes (phylum) presented a significant positive correlation with industrially processed meat consumption.

**Figure 3:**
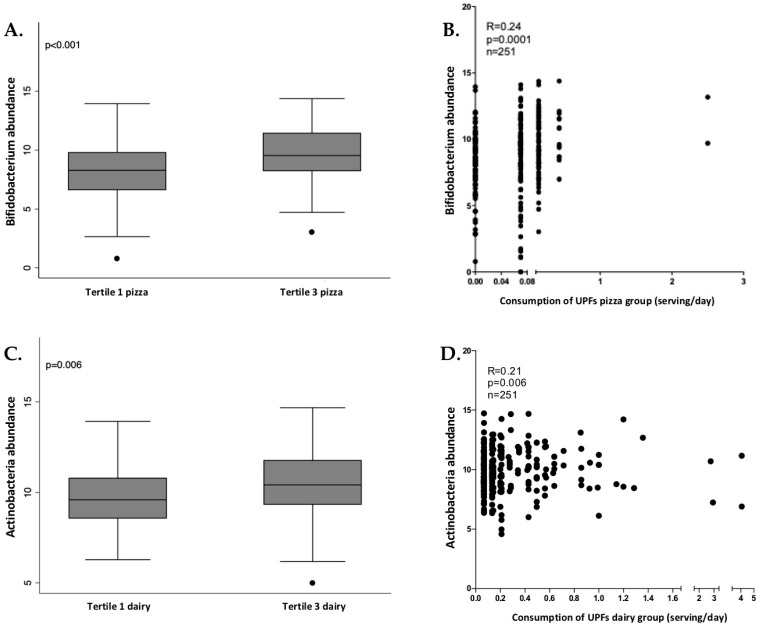
Box plot of differences in *Bifidobacterium* abundance according to tertiles of ultra-processed pizza consumption (**A**) and the correlation analysis (**B**) in women. Box plot of differences in Actinobacteria abundance according to tertiles of ultra-processed dairy consumption (**C**) and the correlation analysis (**D**) in women. The Y axis represents the relative bacterial abundance and the X axis represents the tertiles 1 and 3 of UPFs consumption. Only shown *Bifidobacterium* genus in association with pizza consumption due to the similarities with Bifidobacteriales and Actinobacteria. Differences between tertiles were assessed by Mann-Whitney test.

In women, Actinobacteria phylum, Bifidobacteriales (an order from Actinobacteria) and *Bifidobacterium* (a genus from Bifidobacteriales) presented significant and positive associations with industrially processed dairy products and pizza consumption. The relation between these bacteria and these UPF groups was checked through tertiles of consumption, as shown in Figure 3, showing that the tertile 3 (higher consumption of pizza and dairy) also presented a higher abundance of *Bifidobacterium,* Bifidobacteriales and Actinobacteria.

**Figure 4:**
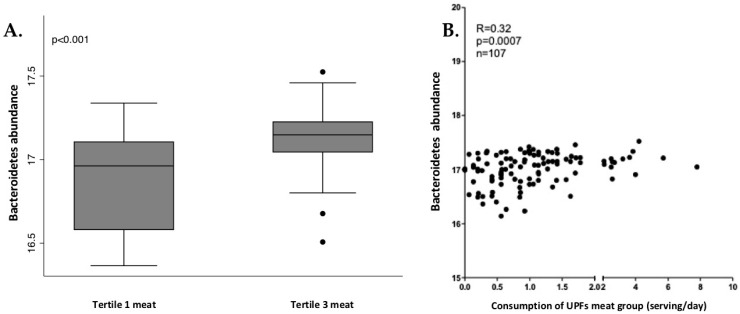
Box plot of differences in Bacteroidetes abundance according to tertiles of industrially processed meat consumption (**A**) and the correlation analysis (**B**) in men. The Y axis represents the relative bacterial abundance and the X axis represents the tertiles 1 and 3 of UPFs consumption. Only shown are Bacteroidetes, due to similarities with Bacteroidia. Differences between tertiles were assessed by Mann-Whitney test.

In men, Bacteroidetes phylum and Bacteroidia class presented significant and positive correlation with industrially processed meat consumption. The relation between these bacteria and industrially processed meat consumption was analyzed through tertiles of consumption, as shown in Figure 4. Tertile 3 (higher consumption) showed a higher abundance of Bacteroidia and Bacteroidetes.

## 4. Discussion

In this study, the effects of UPFs consumption on gut microbiota (with putative consequences on health) was studied in a Spanish population, taking into account the differences between sexes. In recent years, unhealthy diets (characterized by a low intake of legumes, whole grains, nuts, fruits and seafood) are rising around the globe, together with a high intake of industrially processed products. In this regard, the sales of UPFs have hugely increased between 2000 and 2013. Actually, sales of these products in Spain rose by 18.5% during this period [47]. This increase in UPFs consumption has been associated with different health problems, including higher risk of cardiovascular disease, cerebrovascular disease, depression and all-cause of mortality [48].

In our Spanish population, we have observed significant differences in anthropometric and biochemical values according to UPFs consumption. In the whole population, subjects who consumed more than 5 UPFs presented higher values of total energy intake, BMI, weight, waist and hip circumference, fat mass and triglycerides. These results are in line with previous investigations that evidenced the close relationship between UPFs consumption and obesity [49]. As shown in the SUN cohort, participants with a high level of consumption (in the highest quartile) presented a higher risk of developing overweight or obesity compared to participants with low consumption [9]. Participants who consumed more UPFs also presented more cases of depression and anxiety. Positive associations have been found in the literature between UPFs consumption and the risk of depressive symptoms, which are strongest in people with lower exercise levels [11,50,51].

Similarly, total energy intake, weight and hip circumference were significantly higher in the women who consumed more UPFs, showing the positive association between UPFs and the development of obesity. More cases of depression and anxiety were also found in the group of women who consumed more than 5 UPFs. A study in Swiss women also evidenced the association between the UPFs consumption and excessive body weight [52], but more studies are needed to understand the effects of UPFs consumption in women.

In men who consumed more than 5 UPFs, energy intake, BMI and body weight were also significantly higher, but unlike women, they also presented higher values in several biochemical parameters (triglycerides, ALT and TNFα). In this context, the consumption of UPFs has also been linked to an increase of inflammatory status. For example, Nestares et al. (2021) found that a high consumption of UPFs accompanied by low physical activity levels resulted in a worse inflammatory profile [53]. However, studies on the effects of UPFs in male subjects are very scarce.

Interestingly, we found that the energy intake from UPFs was 22.8% in whole population, 21.5% in women and 26.3% in men. The increase in the percentage of energy from UPFs is accompanied by a reduction in the adherence to Mediterranean diet. These finding are in line with a recent study showing that, compared to a diet free from UPFs, a high UPFs diet (with >80% of UPFs) triggered an increase in energy intake of proximate 500 kcal per day. Moreover, this study showed that, in two weeks, participants exposed to the high UPF diet gained 0.9 kg compared to participants exposed to the non-UPF diet, who lost 0.9 kg. The poor nutritional quality of UPFs, the hyper-palatable taste and the use of artificialized matrices with the subsequent effect on satiety feeling have been associated with an increased risk of obesity, which has been attributed to their high content in total fats, saturated fats, and free sugars, [54], and also the use of additives, some of them linked to the deleterious effects of UPFs [55,56]. In this regard, investigations can be found studying additive exposure and long-term potential effects in humans [56].

Moreover, although the impact of diet on microbiota composition has been largely evidenced in the literature, the effects of UPFs on gut microbiota have not been deeply studied.

Dietary modifications can have a direct impact on the gut microbiota diversity and functionality. The environment created in the gut by a high consumption of UPFs, a hallmark of the Western diet, might contribute to the deleterious effects of these foods on low-grade systemic inflammatory and oxidative status and even neurodegenerative diseases [57].

The results of this investigation showed that alpha diversity decreased in men who consumed a higher quantity of UPFs. The diversity and composition of gut microbiota presents a greater degree of affectation in individuals who consume low-fiber diets. Alterations in the diversity and composition of the microbiota may lead to an imbalance in the production of short chain fatty acids and other postbiotics, which contributes to the proinflammatory characteristic of chronic metabolic diseases [58]. No significant results were found in Firmicutes-Bacteroidetes ratio. Although some studies related this ratio with obesity, this finding shows that this is still not clear [59].

The effects of UPF on gut microbiota need more investigation since information about microbiota components and UPF is still scarce. In fact, the results found in females who consumed more than 5 serv/d of adjusted UPFs included groups of bacteria that have no link with UPF in the literature. However, some of these have been studied in relation with Western diet. For example, the genus *Shigella* was found to be positively associated with a Western diet in the pioneer study of de Filippo et al. (2010) [22]. *Parabacteroides* is another genus significantly overrepresented in women who consumed more UPFs. The association of *Parabacteroides* with UPF is not clear but a recent study in adolescents has shown that SSB consumption elevated fecal abundance of *Parabacteroides*, which negatively correlated with memory task performance [60]. Enterobacteriales also increased with the consumption of UPFs in women. This order includes bacteria that have been related with dysbiosis, gut inflammation and the development of inflammatory bowel disease [61].

On the other hand, *Melainabacter* has been described as a beneficial genus for the host due to the production of vitamin K and for the digestion of plant fibers [62]. In women who consumed more UPFs this genus showed a decrease in abundance. Similarly, *Lachnospira* showed a decrease in women who consumed more UPFs. Some publications suggest that this genus increases when the adherence to Mediterranean diet is high [63].

Actinobacteria, Bifidobacteriales and *Bifidobacterium* showed an increase in women who consumed more UPFs. This group of bacteria was also significantly associated with the consumption of industrially processed dairy products and pizza in women. It is well-known that diets high in fermentable carbohydrates are usually associated with higher relative abundance of *Bifidobacterium*, which is capable of degrading polysaccharides, oligosaccharides and sugars. Other food ingredients present in UPFs, such as certain low-calorie sweeteners, can also favor *Bifidobacterium* [64]. Dairy-related Bifidobacteria (belonging to the Actinobacteria phylum) are used in an extensive variety of probiotic dairy products, such as milk, cheese, and frozen dairy products [65]. A high consumption of these products (although some of them are UPFs) may contribute to the increase of *Bifidobacterium*, although more investigations are needed. On the other hand, the relation between the consumption of pizza and higher levels of *Bifidobacterium* is unclear, although the high number of polysaccharides in this food might be related. Moreover, some studies suggest that women present a high abundance of *Bifidobacterium* in comparison with men [66].

In our study, those men who consumed more UPFs presented a higher abundance of *Granulicatella*, a genus that has been previously associated with obesity and *Blautia,* a genus previously associated with fat accumulation [67]. The family Carnobacteriaceae, which has been also related to obesity [68], was overrepresented in men who consumed more UPFs [68]. Men who consumed more than 5 serv/d of UPFs (adjusted) also presented a higher abundance of Bacteroidaceae (family), Bacteroidia (class) and Bacteroidetes (phylum). Some publications suggest that very high-fat diets are able to increase bile-resistant organisms, such as some members of the Bacteroidetes phylum [69]. Nevertheless, the relation between Bacteroidetes and the consumption of industrially processed meat in men is not clear in the literature.

Moreover, the addition of chemicals (not only non-sugar sweeteners, but also emulsifiers, preservatives, colorings and anti-oxidants) to UPF for improving appearance, longevity or taste could interact with the gut microbiota. In this regard, studies in mice have shown that additives such as emulsifiers (carboxymethylcellulose and polysorbate-80) can induce low grade inflammation, destruction of the mucus layer, epithelial encroachment, alteration in species composition (decreasing bacterial diversity) and colitis in wild type mice [70,71].

The reason why some bacteria presented a higher abundance in women and others in men needs more investigation in order to clarify the role of sex in the intricate relationship between diet and microbiota. In this context, an estrogen–gut microbiome axis has been proposed [72]. The gut microbiota regulates estrogen levels through the secretion of β-glucuronidase, an enzyme that deconjugates estrogens into their active forms. It has been proposed that a dysbiosis characterized by lower microbial diversity might impair this process, decreasing deconjugation and reducing circulating estrogen levels. On the other hand, it is known that the composition of the gut microbiota is directly influenced by sex hormones. For example, 17β-estradiol supplementation is able to change gut microbiota diversity and the Firmicutes/Bacteroidetes ratio in male mice [73]; and changes in the sex steroid balance (i.e., circulating estradiol to testosterone ratio) have been linked to altered gut microbiota composition including the Firmicutes to Bacteroidetes ratio [74].

In this context, making healthy foods from raw material available and affordable is essential to reduce the consumption of UPF, which consequently will trigger a positive effect on gut microbiota.

This study has some limitations. The FFQ used was not exactly designed to collect data on UPFs, although most of the well-known UPFs are included. Therefore, some UPFs such as energy bars, energy drinks, meat or vegetable nuggets were not included. Variables were adjusted for possible confounders (age, BMI and total energy intake), but other potential confounders may also have an influence. Our sample is relatively small and the population cannot be completely representative of the general population (only recruited subjects from Navarra, Spain). In addition, the cut-off value chosen in this study was less than 3 servings per day of UPFs and more than 5 in order to obtain two well-defined and balanced groups for carrying out the comparison analyses of the gut microbiota. However, we are aware that results could change depending on the criteria followed for establishing the comparison groups. The lack of references in the literature to gut microbiota and UPFs consumption may also make the choice of an adequate cut-off point difficult.

To the best of our knowledge, this is the first study evaluating the relation between UPFs consumption and microbiota taking into account differences between sexes in a Spanish population. Investigations about the effect of UPFs on gut microbiota taking into account the role of sex are paving the way for the future.

We used validated methods and the analysis was adjusted for potential confounders. Furthermore, the NOVA classification ranks food categories according to the extent and purpose of food processing, instead of in terms of nutrients. It is a recognized and useful classification for conducting nutritional research [6].

## 5. Conclusions

This study suggests that a consumption higher than five servings per day of UPF may affect gut microbiota composition differently in women and men. This work evidences that the consumption of UPFs may affect gut microbiota composition in a different manner depending on sex, which might be a mechanism involved in the risk for different diseases. We also evidenced that some bacteria were associated with specific groups of UPFs. However, further research is needed to confirm these observations.

## Figures and Tables

**Table 1:** Characteristics of the participants according to UPFs consumption categorized according to <3 servings per day and >5 servings per day.

	Whole Population			Women			Men			*p* Value (Women-Men < 3) ^4^	*p* Value (Women-Men > 5) ^5^
Variables	<3 serv/d (*n* = 96)	>5 serv/d (*n* = 90)	*p* Value ^1^	<3 serv/d (*n* = 57)	>5 serv/d (*n* = 66)	*p* Value ^2^	<3 serv/d (*n* = 39)	>5 serv/d (*n* = 24)	*p* Value ^3^		
**UPF consumption (serv/ d)**	2.0 ± 0.1	6.4 ± 0.2	**<0.001**	2.0 ± 0.1	6.2 ± 0.1	**<0.001**	2.0 ± 0.1	7.01 ± 0.52	**<0.001**	0.76	0. 21
**Age (y)**	46 ± 1	43 ± 1	**0.03**	45 ± 1	43.5± 1	0.17	48 ± 1	43.5 ± 1.9	0.08	0.27	0.98
**Smoking**	20	24	0.54	11	15	0.68	9	9	0.98	0.47	0.23
**Alcohol habit**	61	51	0.36	30	30	0.93	30	21	0.74	0.98	0.16
**METs**	28.5 ± 1.5	22.5 ± 2.4	0.07	22.6 ± 2.5	18.1 ± 1.9	0.15	37.0 ± 3.8	34.5 ± 6.7	0.72	**<0.001**	**0.007**
**Depression prevalence**	0	6	**0.01**	0	5	**0.01**	0	1	0.43	0.89	0.19
**Anxiety prevalence**	4	8	**0.01**	3	6	**0.02**	1	2	0.53	0.36	0.14
**Energy intake (kcal)**	2444 ± 50	3685 ± 97	**<0.001**	2372 ± 61	3608 ± 107	**<0.001**	2629 ± 83	3844 ± 195	**<0.001**	**0.02**	0.25
**Energy from UPFs (%)**	10.1 ± 0.5	22.8 ± 1.1	**<0.001**	8.7 ± 0.4	21.5 ± 1.3	**<0.001**	12.1 ± 0.8	26.3 ± 2.0	**<0.001**	**0.002**	0.06
**Adherence to MD**	8.0 ± 0.1	6.0 ± 0.1	**<0.001**	7.0 ± 0.2	6.0 ± 0.2	**<0.001**	7.0 ± 0.2	6.0 ± 0.3	**0.006**	0.77	0.78
**BMI baseline (kg/m^2^)**	29.2 ± 0.4	30.9 ± 0.4	**0.02**	29.5 ± 0.5	30.5 ± 0.6	0.19	29.5 ± 0.6	31.6 ± 0.6	**0.02**	0.99	0.25
**Weight (kg)**	81.1 ± 1.2	87.1 ± 1.5	**0.002**	77.5 ± 1.3	82.0 ± 1.7	**0.04**	90.6 ± 2.2	97.6 ± 2.2	**0.03**	**<0.001**	**<0.001**
**Waist circumference (cm)**	96 ± 1	101 ± 1	**0.02**	94 ± 1	97 ± 2	0.14	102 ± 2	108 ± 2	0.07	**0.01**	**0.002**
**Hip circumference (cm)**	108 ± 1	111 ± 1	**0.01**	108 ± 1	112 ± 1	**0.03**	106 ± 1	108 ± 1	0.13	**0.11**	0.05
**SBP (mmHg)**	126 ± 1	125 ± 2	0.71	122 ± 2	120 ± 2	0.33	135 ± 3	136 ± 3	0.81	**0.001**	**<0.001**
**DBP (mmHg)**	78 ± 1	78 ± 1	0.86	76 ± 1	76 ± 1	0.91	84 ± 2	82 ± 2	0.44	**<0.001**	**0.002**
**Fat mass (kg)**	28.6 ± 1.3	33.8 ± 1.4	**0.01**	29.2 ± 1.6	34.1 ± 1.9	0.05	27.2 ± 2.3	33.2 ± 2.0	0.06	0.51	0.75
**Visceral fat mass (kg)**	1.2 ± 0.08	1.3 ± 0.09	0.22	0.9 ± 0.06	1.0 ± 0.08	0.45	1.9 ± 0.2	2.1 ± 0.1	0.58	**<0.001**	**<0.001**
**Glucose (mg/dL)**	95 ± 1	94 ± 1	0.74	94 ± 2	92 ± 1	0.49	97 ± 2	98 ± 2	0.71	0.22	**0.006**
**Total cholesterol (mg/dL)**	211 ± 2	215 ± 4	0.44	211 ± 4	213 ± 5	0.77	210 ± 6	218 ± 6	0.34	0.89	0.51
**HDL-cholesterol (mg/dL)**	59 ± 1	55 ± 1	**0.04**	61 ± 1	59 ± 2	0.41	52 ± 1	46 ± 1	**0.01**	**<0.001**	**<0.001**
**LDL-cholesterol (mg/dL)**	64 ± 3	57 ± 4	0.22	63 ± 4	57 ± 5	0.37	67 ± 7	58 ± 6	0.35	0.59	0.92
**Triglycerides (mg/dL)**	86 ± 3	105 ± 6	**0.004**	84 ± 4	92 ± 5	0.23	92 ± 7	130 ± 12	**0.006**	0.29	**0.001**
**ALT (U/L)**	22 ± 1	23 ± 1	0.44	20 ± 1	19 ± 1	0.59	26 ± 2	31 ± 2	**0.04**	**0.04**	**<0.001**
**AST (U/L)**	22 ± 1	21 ± 1	0.57	21 ± 1	20 ± 1	0.33	24 ± 1	24 ± 1	0.72	0.14	**<0.001**
**Insulin (mU/L)**	7.3 ± 0.4	8.0 ± 0.4	0.36	7.5 ± 0.5	7.4 ± 0.5	0.93	7.0 ± 0.7	9.0 ± 1	0.09	0.59	0.13
**Adiponectin (µg/mL)**	12.3 ± 0.4	11.5 ± 0.4	0.21	13.7 ± 0.5	12.9 ± 0.5	0.35	8.9 ± 0.5	8.6 ± 0.5	0.67	**<0.001**	**<0.001**
**TNF (pg/mL)**	0.8 ± 0.02	0.9 ± 0.03	0.16	0.8 ± 0.03	0.8 ± 0.04	0.95	0.8 ± 0.02	1.0 ± 0.06	**0.009**	0.99	**0.01**
**Leptin (ng/mL)**	32.3 ± 2.2	35.1 ± 2.8	0.44	39.9 ± 2.6	45.3 ± 3.6	0.22	12.6 ± 1.6	14.1 ± 1.7	0.52	**<0.001**	**<0.001**
**HOMA-IR**	1.8 ± 0.1	1.8 ± 0.1	0.69	1.8 ± 0.2	1.7 ± 0.1	0.61	1.7 ± 0.2	2.2 ± 0.2	0.15	0.75	0.06
**CRP (µg/mL)**	2.4 ± 0.3	2.8 ± 0.3	0.31	2.6 ± 0.3	3.1 ± 0.4	0.41	1.8 ± 0.4	2.3 ± 0.3	0.41	0.21	0.24

Variables are expressed as mean ± SE for quantitative variables and as numbers of cases for qualitative variables. Differences between groups were assessed by *t*-test or Mann–Whitney test (METs, glucose, adiponectin, ALT, AST, TNFa, HOMA-IR and CRP) according to the distribution of data and quantitative values variables were assessed by Chi-squared. Serv/day: servings per day. ALT: alanine aminotransferase, AST: aspartate aminotransferase, BMI: body mass index, CRP: C-reactive protein, DBP: diastolic blood pressure, HDL-c: hihg density lipoprotein cholesterol, HOMA-IR: homeostatic model assessment for insulin resistance index, LDL-c: low density lipoprotein cholesterol, MD: Mediterranean diet, MET: metabolic equivalent of tasks, SBP: systolic blood pressure, Serv/d: servings per day, TNF: tumor necrosis factor alpha, TSH: thyroid-stimulating hormone, UPF: ultra-processed food. Each variable was analyzed using total UPFs consumption adjusted for BMI, age and energy intake, except differences in age, BMI, weight, energy form UPFs and total energy. *p* values were obtained using *t*-test or Mann-Whitney test (according to the normality of the data) for quantitative variables and chi square for qualitative variables. ^1^ *p* value of the comparison between subjects who consumed less than 3 servings per day of UPF and more than 5 in whole population. ^2^ *p* value of the comparison between women who consumed less than 3 servings per day of UPF and more than 5. ^3^ *p* value of the comparison between men who consumed less than 3 servings per day of UPF and more than 5. ^4^ *p* value of the comparison between women and men who consumed less than 3 servings per day of UPF. ^5^ *p* value of the comparison between women and men who consumed more than 5 servings per day of UPF. Significant values are in bold type.

**Table 2:** Servings per day of the different groups of UPF separated by sex.

	Women			Men			Women-Men < 3	Women-Men > 5
Servings/day	<3 serv/d	>5 serv/d	*p* Value ^1^	<3 serv/d	>5 serv/d	*p* Value ^2^	*p* Value ^3^	*p* Value ^4^
**Dairy consumption**	0.1 ± 0.01	0.3 ± 0.08	**<0.001**	0.23 ± 0.04	0.16 ± 0.02	**<0.001**	0.98	0.94
**Meat consumption**	0.7 ± 0.03	1.4 ± 0.1	**<0.001**	0.7 ± 0.2	1.2 ± 0.1	**<0.001**	0.61	**0.02**
**Cereals consumption**	0.02 ± 0.01	0.06 ± 0.02	0.28	0.13 ± 0.08	0.24± 0.12	0.21	0.69	0.34
**Pizza consumption**	0.07 ± 0.007	0.2 ± 0.05	**<0.001**	0.1 ± 0.01	0.08 ± 0.02	0.64	0.31	0.09
**Margarine consumption**	0.05 ± 0.01	0.1 ± 0.04	0.48	0.16 ± 0.07	0.06 ± 0.03	**0.005**	0.07	0.58
**Fried consumption**	0.1 ± 0.009	0.2 ± 0.03	**<0.001**	0.13 ± 0.03	0.24 ± 0.02	**0.001**	0.33	0.32
**Cookies consumption**	0.1 ± 0.02	0.7 ± 0.1	**<0.001**	0.36 ± 0.15	0.76 ± 0.07	**<0.001**	0.59	0.29
**Light products consumption**	0.07 ± 0.01	0.6 ± 0.1	**<0.001**	0.07 ± 0.13	0.29 ± 0.03	0.06	0.96	0.66
**Ready-to-eat food consumption**	0.05 ± 0.006	0.08 ± 0.02	0.26	0.03 ± 0.009	0.08 ± 0.02	**0.009**	0.88	0.79
**Mayonnaise consumption**	0.05 ± 0.005	0.1 ± 0.02	**0.04**	0.08 ± 0.03	0.13 ± 0.02	**0.02**	0.61	0.11
**Alcohol consumption**	0.03 ± 0.008	0.03 ± 0.009	0.98	0.11 ± 0.03	0.22 ± 0.06	0.15	**0.002**	**<0.001**
**Pastries consumption**	0.7 ± 0.05	2.4 ± 0.3	**<0.001**	0.85 ± 0.15	2.2 ± 0.31	**<0.001**	0.13	0.23
**SSB consumption**	0.09 ± 0.01	0.3 ± 0.07	**<0.001**	0.19 ± 0.05	0.43 ± 0.13	**<0.001**	0.16	**<0.001**

Variables are expressed as mean ± SE. Differences between groups were assessed by Mann–Whitney test due to the non-parametric distribution of data. Serv/d: servings per day. Groups of UPFs refer to industrially processed dairy, meat, pizza, fried food, cookies, pastries and breakfast cereals. SSB: industrially processed sugar-sweetened beverages. *p* values were obtained using *t*-test or Mann-Whitney test (according to the normality of the data). ^1^ *p* value of the comparison between women who consumed less than 3 servings per day of UPF and more than 5. ^2^ *p* value of the comparison between men who consumed less than 3 servings per day of UPF and more than 5. ^3^ *p* value of the comparison between women and men who consumed less than 3 servings per day of UPF. ^4^ *p* value of the comparison between women and men who consumed more than 5 servings per day of UPF. Significant values in bold type.

**Table 3:** Bacterial taxa significantly different between subjects who consumed less than 3 serv/d of adjusted UPFs and more than 5 (from genus to phylum) analyzed by EdgeR.

Bacteria Name	Log2FC	*p* Value	FDR
**Genus**			
***Gemmiger***	2.163	1.1 × 10^−9^	7.11 × 10^−8^
***Granulicatella***	1.759	6.4 × 10^−7^	1.98 × 10^−5^
***Parabacteroides***	0.969	1.9 × 10^−4^	0.002
***Shigella***	1.622	5.6 × 10^−4^	0.008
***Bifidobacterium***	1.075	7.0 × 10^−4^	0.008
***Anaerofilum***	0.786	0.001	0.01
***Lachsnopira***	−1.034	0.003	0.02
***Roseburia***	−0.746	0.003	0.02
***Cc_115***	0.777	0.007	0.04
***Oxalobacter***	1.055	0.008	0.04
***Collinsella***	0.735	0.008	0.04
**Family**			
**Carnobacteriacea**	1.772	4.69 × 10^−7^	1.54 × 10^−5^
**Oxalobacteraceae**	1.324	6.59 × 10^−4^	0.01
**Bifidobacteriaceae**	0.919	0.003	0.03
**Order**			
**Bifidobacteriales**	1.125	3.81 × 10^−4^	0.006
**Pasteurellales**	−1.180	0.005	0.04
**Class**			
**Actinobacteria**	0.852	8.86 × 10^4^	0.01
**Phylum**			
**Actinobacteria**	0.852	8.86 × 10^−4^	0.01

Log2FC: logarithm 2 fold change (positive value when the abundance increases in group of consumption >5 serv/d of adjusted UPFs); FDR: False Discovery Rate.

**Table 4:** Bacterial taxa significantly different between women who consumed less than 3 serv/d of adjusted UPFs and more than 5 (from genus to phylum) analyzed by EdgeR.

Bacterial Name	Log2FC	*p* Value	FDR
**Genus**			
***Acidaminococcus***	4.022	4.92 × 10^−9^	3.0 × 10^−7^
***Butyrivibrio***	2.899	4.17 × 10^−7^	1.3 × 10^−5^
***Gemmiger***	2.34	6.25 × 10^−7^	1.3 × 10^−5^
***Shigella***	2.171	2.14 × 10^−4^	0.003
***Anaerofilum***	1.228	3.4 × 10^−4^	0.004
***Parabacteroides***	1.018	0.002	0.02
***Melainabacter***	−1.976	0.002	0.02
***Lachnospira***	−1.321	0.003	0.02
***Bifidobacterium***	1.052	0.006	0.04
**Order**			
**Enterobacteriales**	1.682	0.002	0.03
**Bifidobacteriales**	1.079	0.004	0.03
**Phylum**			
**Actinobacteria**	0.860	0.006	0.04

Log2FC: logarithm 2 fold change (positive value when the abundance increases in group of consumption >5 serv/d of adjusted UPFs); FDR: False Discovery Rate.

**Table 5:** Bacterial taxa significantly different between men who consumed less than 3 serv/d of adjusted UPF and more than 5 (from genus to phylum) analyzed by EdgeR.

Bacterial Name	log2FC	*p* Value	FDR
**Genus**			
***Anaerostipes***	−4.361	3.04 × 10^−7^	1.88 × 10^−5^
***Granullicatella***	3.019	7.94 ×10^−6^	2.46 × 10^−4^
***Blautia***	1.231	0.002	0.04
**Family**			
**Carnobacteriaceae**	2.71	2.2 × 10^−5^	7.23 × 10^−4^
**Clostridiaceae**	−1.313	0.002	0.03
**Bacteroidaceae**	1.023	0.002	0.03
**Peptostreptococcaceae**	1.443	0.005	0.04
**Class**			
**Bacteroidia**	0.804	7.37 × 10^−4^	0.01
**Phylum**			
**Bacteroidetes**	0.799	1.1 × 10^−4^	8.84 × 10^−4^

Log2FC: logarithm 2 fold change (positive value when the abundance increases in group of consumption >5 serv/d of adjusted UPFs); FDR: False Discovery Rate.

## Data Availability

The data presented in this study are available on request from the corresponding author. Sequenced data submitted to the NCBI SRA repository under the accession number PRJNA623853.

## Supplementary Materials

The following are available online at https://www.mdpi.com/article/10.3390/nu13082710/s1. Table S1: Ultra-processed food (and subclassification) included in the food-frequency questionnaire used for this study. Supplementary Table S2. Baseline anthropometric and biochemical characteristic of the participant excluded for microbiota analysis (subjects who consumed between 3 and 5 servings per day of UPFs). Supplementary Table S3. Non-significant bacterial taxa (FDR > 0.05) analyzed by EdgeR between subjects who consumed less than 3 and more than 5 servings per day of UPFs. Supplementary Table S4. Non-significant bacterial taxa (FDR > 0.05) analyzed by EdgeR between women who consumed less than 3 and more than 5 servings per day of UPFs. Supplementary Table S5. Non-significant bacterial taxa (FDR > 0.05) analyzed by EdgeR between women who consumed less than 3 and more than 5 servings per day of UPFs.

## References

[1] Hasan N., Yang H. (2019). Factors affecting the composition of the gut microbiota, and its modulation. PeerJ.

[2] Singh R.K., Chang H.W., Yan D., Lee K.M., Ucmak D., Wong K., Abrouk M., Farahnik B., Nakamura M., Zhu T.H. (2017). Influence of diet on the gut microbiome and implications for human health. J. Transl. Med..

[3] Koponen K.K., Salosensaari A., Ruuskanen M.O., Havulinna A.S., Männistö S., Jousilahti P., Palmu J., Salido R., Sanders K., Brennan C. (2021). Associations of healthy food choices with gut microbiota profiles. Am. J. Clin. Nutr..

[4] Rauber F., Steele E.M., da Costa Louzada M.L., Millett C., Monteiro C.A., Levy R.B. (2020). Ultra-processed food consumption and indicators of obesity in the United Kingdom population (2008–2016). PLoS ONE.

[5] Fardet A., Rock E. (2020). Ultra-processed foods and food system sustainability: What are the links?. Sustainability.

[6] Monteiro C.A., Cannon G., Levy R.B., Moubarac J.C., Louzada M.L.C., Rauber F., Khandpur N., Cediel G., Neri D., Martinez-Steele E. (2019). Ultra-processed foods: What they are and how to identify them. Public Health Nutr..

[7] Marti A. (2019). Ultra-processed foods are not “real food” but really affect your health. Nutrients.

[8] De Deus Mendonça R., Souza Lopes A.C., Pimenta A.M., Gea A., Martinez-Gonzalez M.A., Bes-Rastrollo M. (2017). Ultra-processed food consumption and the incidence of hypertension in a mediterranean cohort: The seguimiento universidad de navarra project. Am. J. Hypertens..

[9] Bhurosy T., Kaschalk E., Smiley A., He K. (2017). Comment on “ultraprocessed food consumption and risk of overweight and obesity: The University of Navarra Follow-Up (SUN) cohort study”. Am. J. Clin. Nutr..

[10] Martínez Steele E., Juul F., Neri D., Rauber F., Monteiro C.A. (2019). Dietary share of ultra-processed foods and metabolic syndrome in the US adult population. Prev. Med..

[11] Gómez-Donoso C., Sánchez-Villegas A., Martínez-González M.A., Gea A., Mendonça R.d.D., Lahortiga-Ramos F., Bes-Rastrollo M. (2020). Ultra-processed food consumption and the incidence of depression in a Mediterranean cohort: The SUN Project. Eur. J. Nutr..

[12] Srour B., Fezeu L.K., Kesse-Guyot E., Allès B., Debras C., Druesne-Pecollo N., Chazelas E., Deschasaux M., Hercberg S., Galan P. (2020). Ultraprocessed Food Consumption and Risk of Type 2 Diabetes among Participants of the NutriNet-Santé Prospective Cohort. JAMA Intern. Med..

[13] Senghor B., Sokhna C., Ruimy R., Lagier J.C. (2018). Gut microbiota diversity according to dietary habits and geographical provenance. Hum. Microbiome J..

[14] Cuevas-Sierra A., Riezu-Boj J.I., Guruceaga E., Milagro F.I., Martínez J.A. (2020). Sex-Specific Associations between Gut Prevotellaceae and Host Genetics on Adiposity. Microorganisms.

[15] Monda V., Villano I., Messina A., Valenzano A., Esposito T., Moscatelli F., Viggiano A., Cibelli G., Chieffi S., Monda M. (2017). Exercise modifies the gut microbiota with positive health effects. Oxid. Med. Cell. Longev..

[16] Mangiola F., Nicoletti A., Gasbarrini A., Ponziani F.R. (2018). Gut microbiota and aging. Eur. Rev. Med. Pharmacol. Sci..

[17] Santos-Marcos J.A., Haro C., Vega-Rojas A., Alcala-Diaz J.F., Molina-Abril H., Leon-Acuña A., Lopez-Moreno J., Landa B.B., Tena-Sempere M., Perez-Martinez P. (2019). Sex differences in the gut microbiota as potential determinants of gender predisposition to disease. Mol. Nutr. Food Res..

[18] Yurkovetskiy L., Burrows M., Khan A.A., Graham L., Volchkov P., Becker L., Antonopoulos D., Umesaki Y., Chervonsky A.V. (2013). Gender Bias in Autoimmunity is Influenced by Microbiota. Immunity.

[19] Markle J.G.M., Frank D.N., Mortin-Toth S., Robertson C.E., Feazel L.M., Rolle-Kampczyk U., Von Bergen M., McCoy K.D., Macpherson A.J., Danska J.S. (2013). Sex differences in the gut microbiome drive hormone-dependent regulation of autoimmunity. Science.

[20] Org E., Mehrabian M., Parks B.W., Shipkova P., Liu X., Drake T.A., Lusis A.J. (2016). Sex differences and hormonal effects on gut microbiota composition in mice. Gut Microbes..

[21] Lay C., Rigottier-Gois L., Holmstrøm K., Rajilic M., Vaughan E.E., De Vos W.M., Collins M.D., Thiel R., Namsolleck P., Blaut M. (2005). Colonic microbiota signatures across five northern European countries. Appl. Environ. Microbiol..

[22] De Filippo C., Cavalieri D., Di Paola M., Ramazzotti M., Poullet J.B., Massart S., Collini S., Pieraccini G., Lionetti P. (2010). Impact of diet in shaping gut microbiota revealed by a comparative study in children from Europe and rural Africa. Proc. Natl. Acad. Sci. USA.

[23] Asarian L., Geary N. (2013). Sex differences in the physiology of eating. Am. J. Physiol. Regul. Integr. Comp. Physiol..

[24] Luzia Da Silva C., Sousa A.G., Pereira L., Leão Borges S., Macedo Da Costa T.H. (2021). Usual consumption of ultra-processed foods and its association with sex, age, physical activity, and body mass index in adults living in Brasília City, Brazil. Rev. Bras. Epidemiol..

[25] Ramos-Lopez O., Riezu-Boj J.I., Milagro F.I., Goni L., Cuervo M., Martinez J.A. (2018). Differential lipid metabolism outcomes associated with ADRB2 gene polymorphisms in response to two dietary interventions in overweight/obese subjects. Nutr. Metab. Cardiovasc. Dis..

[26] Goni L., Riezu-Boj J.I., Milagro F.I., Corrales F.J., Ortiz L., Cuervo M., Martínez J.A. (2018). Interaction between an ADCY3 genetic variant and two weight-lowering diets affecting body fatness and body composition outcomes depending on macronutrient distribution: A randomized trial. Nutrients.

[27] World Medical Association (2014). World Medical Association Declaration of Helsinki: Ethical principles for medical research involving human subjects. J. Am. Coll. Dent..

[28] Martin-Moreno J.M., Boyle P., Gorgojo L., Maisonneuve P., Fernandez-Rodriguez J.C., Salvini S., Willett W.C. (1993). Development and Validation of a Food Frequency Questionnaire in Spain. Int. J. Epidemiol..

[29] Moreiras O., Carbajal A., Cabrera L., Cuadrado C. (2011). Tablas de Composicion de Alimentos (Ciencia y Tecnica).

[30] Cranston J.M., Crockett A.J., Moss J.R., Pegram R.W., Stocks N.P. (2020). Ultra-Processed Food and Health Outcomes: A narrative review. Nutrients.

[31] Monteiro C.A., Cannon G., Levy R., Moubarac J.-C., Jaime P., Martins A.P., Canella D., Louzada M., Parra D. (2016). NOVA. The star shines bright. World Nutr..

[32] Morea N. Ultra-Processed Foods: Nova Classification | Food Compliance Solutions. https://regulatory.mxns.com/en/ultra-processed-foods-nova-classification.

[33] Ojeda-Rodríguez A., Zazpe I., Alonso-Pedrero L., Zalba G., Guillen-Grima F., Martinez-Gonzalez M.A., Marti A. (2020). Association between diet quality indexes and the risk of short telomeres in an elderly population of the SUN project. Clin. Nutr..

[34] Hildebrand F., Tadeo R., Voigt A.Y., Bork P., Raes J. (2014). LotuS: An efficient and user-friendly OTU processing pipeline. Microbiome.

[35] Edgar R.C. (2013). UPARSE: Highly accurate OTU sequences from microbial amplicon reads. Nat. Methods.

[36] Edgar R.C. (2016). UCHIME2: Improved chimera prediction for amplicon sequencing. bioRxiv.

[37] Rideout J.R., He Y., Navas-Molina J.A., Walters W.A., Ursell L.K., Gibbons S.M., Chase J., McDonald D., Gonzalez A., Robbins-Pianka A. (2014). Subsampled open-reference clustering creates consistent, comprehensive OTU definitions and scales to billions of sequences. PeerJ.

[38] Ritari J., Salojärvi J., Lahti L., de Vos W.M. (2015). Improved taxonomic assignment of human intestinal 16S rRNA sequences by a dedicated reference database. BMC Genom..

[39] Gentleman R.C., Carey V.J., Bates D.M., Bolstad B., Dettling M., Dudoit S., Ellis B., Gautier L., Ge Y., Gentry J. (2004). Bioconductor: Open software development for computational biology and bioinformatics. Genome Biol..

[40] World Health Organization Body Mass Index. https://www.euro.who.int/en/health-topics/disease-prevention/nutrition/a-healthy-lifestyle/body-mass-indexbmi#:~:text=BMI%2C%20formerly%20called%20the%20Quetelet,have%20a%20BMI%20of%2022.9.

[41] Whitworth J.A., Chalmers J. (2004). World Health Organisation-International Society of Hypertension (WHO/ISH) hypertension guidelines. Clin. Exp. Hypertens..

[42] Trichopoulou A., Costacou T., Bamia C., Trichopoulos D. (2003). Adherence to a Mediterranean Diet and Survival in a Greek Population. N. Engl. J. Med..

[43] Martínez-González M.A., López-Fontana C., Varo J.J., Sánchez-Villegas A., Martinez J.A. (2005). Validation of the Spanish version of the physical activity questionnaire used in the Nurses’ Health Study and the Health Professionals’ Follow-up Study. Public Health Nutr..

[44] Friedewald W.T., Levy R.I., Fredrickson D.S. (1972). Estimation of the Concentration of Low-Density Lipoprotein Cholesterol in Plasma, Whithout Use of the Preparative Ultracentrifuge. Clin. Chem..

[45] Matthews D.R., Hosker J.P., Rudenski A.S., Naylor B.A., Treacher D.F., Turner R.C. (1985). Homeostasis model assessment: Insulin resistance and β-cell function from fasting plasma glucose and insulin concentrations in man. Diabetologia.

[46] Dhariwal A., Chong J., Habib S., King I.L., Agellon L.B., Xia J. (2017). MicrobiomeAnalyst: A web-based tool for comprehensive statistical, visual and meta-analysis of microbiome data. Nucleic Acids Res..

[47] Pan American Health Organization of the World Health Organization (2015). Ultra-Processed Food and Drink Products in Latin America: Trends, Impact on Obesity, Policy Implications.

[48] Pagliai G., Dinu M., Madarena M.P., Bonaccio M., Iacoviello L., Sofi F. (2020). Consumption of ultra-processed foods and health status: A systematic review and meta-analysis. Br. J. Nutr..

[49] Rauber F., Chang K., Vamos E.P., da Costa Louzada M.L., Monteiro C.A., Millett C., Levy R.B. (2021). Ultra-processed food consumption and risk of obesity: A prospective cohort study of UK Biobank. Eur. J. Nutr..

[50] Adjibade M., Julia C., Allès B., Touvier M., Lemogne C., Srour B., Hercberg S., Galan P., Assmann K.E., Kesse-Guyot E. (2019). Prospective association between ultra-processed food consumption and incident depressive symptoms in the French NutriNet-Santé cohort. BMC Med..

[51] Zheng L., Sun J., Yu X., Zhang D. (2020). Ultra-Processed Food Is Positively Associated With Depressive Symptoms Among United States Adults. Front. Nutr..

[52] Pestoni G., Habib L., Reber E., Rohrmann S., Staub K., Stanga Z., Faeh D. (2021). Ultraprocessed Food Consumption is Strongly and Dose-Dependently Associated with Excess Body Weight in Swiss Women. Obesity.

[53] Nestares T., Martín-Masot R., Flor-Alemany M., Bonavita A., Maldonado J., Aparicio V.A. (2021). Influence of Ultra-Processed Foods Consumption on Redox Status and Inflammatory Signaling in Young Celiac Patients. Nutrients.

[54] Hall K.D., Ayuketah A., Brychta R., Cai H., Cassimatis T., Chen K.Y., Chung S.T. (2019). Ultra-Processed Diets Cause Excess Calorie Intake and Weight Gain: An Inpatient Randomized Controlled Trial of Ad Libitum Food Intake. Cell Metab..

[55] Ivancovsky-Wajcman D., Fliss-Isakov N., Webb M., Bentov I., Shibolet O., Kariv R., Zelber-Sagi S. (2021). Ultra-processed food is associated with features of metabolic syndrome and non-alcoholic fatty liver disease. Liver Int..

[56] Srour B., Touvier M. (2021). Ultra-processed foods and human health: What do we already know and what will further research tell us?. EClinicalMedicine.

[57] Agus A., Denizot J., Thévenot J., Martinez-Medina M., Massier S., Sauvanet P., Bernalier-Donadille A., Denis S., Hofman P., Bonnet R. (2016). Western diet induces a shift in microbiota composition enhancing susceptibility to Adherent-Invasive *E. coli* infection and intestinal inflammation. Sci. Rep..

[58] Nogal A., Valdes A.M., Menni C. (2021). The role of short-chain fatty acids in the interplay between gut microbiota and diet in cardio-metabolic health. Gut Microbes.

[59] Magne F., Gotteland M., Gauthier L., Zazueta A., Pesoa S., Navarrete P., Balamurugan R. (2020). The firmicutes/bacteroidetes ratio: A relevant marker of gut dysbiosis in obese patients?. Nutrients.

[60] Noble E.E., Olson C.A., Davis E., Tsan L., Chen Y.-W., Schade R., Liu C., Suarez A., Jones R.B., Goran M.I. (2020). The gut microbiome regulates memory function. bioRxiv.

[61] Baldelli V., Scaldaferri F., Putignani L., Del Chierico F. (2021). The role of enterobacteriaceae in gut microbiota dysbiosis in inflammatory bowel diseases. Microorganisms.

[62] Di Rienzi S.C., Sharon I., Wrighton K.C., Koren O., Hug L.A., Thomas B.C., Goodrich J.K., Bell J.T., Spector T.D., Banfield J.F. (2013). The human gut and groundwater harbor non-photosynthetic bacteria belonging to a new candidate phylum sibling to Cyanobacteria. eLife.

[63] Shively C.A., Register T.C., Appt S.E., Clarkson T.B., Uberseder B., Clear K.Y.J., Wilson A.S., Chiba A., Tooze J.A., Cook K.L. (2018). Consumption of Mediterranean versus Western Diet Leads to Distinct Mammary Gland Microbiome Populations HHS Public Access. Cell Rep..

[64] Dahl W.J., Rivero Mendoza D., Lambert J.M. (2020). Diet, nutrients and the microbiome. Prog. Mol. Biol. Transl. Sci..

[65] Roy D. (2005). Technological aspects related to the use of bifidobacteria in dairy products. Lait.

[66] Vemuri R., Sylvia K.E., Klein S.L., Forster S.C., Plebanski M., Eri R., Flanagan K.L. (2019). The microgenderome revealed: Sex differences in bidirectional interactions between the microbiota, hormones, immunity and disease susceptibility HHS Public Access. Semin. Immunopathol..

[67] Ozato N., Saito S., Yamaguchi T., Katashima M., Tokuda I., Sawada K., Katsuragi Y., Kakuta M., Imoto S., Ihara K. (2019). *Blautia* genus associated with visceral fat accumulation in adults 20–76 years of age. NPJ Biofilms Microbiomes.

[68] Yang Y., Cai Q., Zheng W., Steinwandel M., Blot W.J., Shu X.-O., Long J. (2019). Oral microbiome and obesity in a large study of low-income and African-American populations. J. Oral Microbiol..

[69] Lane M., Howland G., West M., Hockey M., Marx W., Loughman A., O’Hely M., Jacka F., Rocks T. (2020). The effect of ultra-processed very low-energy diets on gut microbiota and metabolic outcomes in individuals with obesity: A systematic literature review. Obes. Res. Clin. Pract..

[70] White L.S., Van den Bogaerde J., Kamm M. (2018). The gut microbiota: Cause and cure of gut diseases. Med. J. Aust..

[71] Chassaing B., Koren O., Goodrich J.K., Poole A.C., Srinivasan S., Ley R.E., Gewirtz A.T. (2015). Dietary emulsifiers impact the mouse gut microbiota promoting colitis and metabolic syndrome. Naure.

[72] Baker J.M., Al-Nakkash L., Herbst-Kralovetz M.M. (2017). Estrogen–gut microbiome axis: Physiological and clinical implications. Maturitas.

[73] Song C.-H., Kim N., Hee Nam R., In Choi S., Lee H.-N., Surh Y.-J. (2020). 7β-estradiol supplementation changes gut microbiota diversity in intact and colorectal cancer-induced icR male mice. Sci. Rep..

[74] Martinez-Chacon G., Munukka E., Kumar H., Pietila S., Saarinen N., Toivonen R., Salminen S., Hanninen A., Strauss L., Makela S. (2017). A link between sex hormones, obesity and gut microbiota. Endocr. Abstr..

