# Supplementary material for: Gut Microbiota Differences According to Ultra-Processed Food Consumption in a Spanish Population
Source: Nutrients. 2021 Aug 6;13(8):2710. doi: 10.3390/nu13082710 (PMC8398738; doi:10.3390/nu13082710)
Supplement: Supplementary file 1 [file nutrients-13-02710-s001.zip › nutrients-1323414-supplementary.pdf]

Supplementary Table S1. Ultraprocessed food (and subclassification) included in the food-frequency questionnaire used for this study.

Ultraprocessed food groups	Food-frequency questionnaire
**Dairy products**	Packaged cream, smoothies, milk drinks with or without flavors, *petit-suisse*, custard, "fruit" yogurts, flan, pudding, ice-creams
**Meat products**	Ham, smoked bacon, chorizo, salami, mortadella, sausage, hamburger, pate, *foie-gras*, spicy sausage, black pudding mortadella and meatballs
**Cereals**	Packaged breakfast cereals
**Pizza**	Pre-prepared and ready-to-heat pizzas
**Margarine**	Margarine
**Fried products**	Potato chips and fried snacks
**Cookies**	Packaged cookies and chocolate cookies
**Light products**	Slimming products
**Ready-to-eat products**	Instant soups and creams, instant pasta, croquettes and powdered soups and purees
**Mayonnaise**	Mayonnaise sauce
**Alcohol beverages**	Alcoholic drinks produced by fermentation followed by distillation such as whisky, gin, rum
**Pastries**	Packaged buns, pre-prepared pies, prepared cakes, muffins, doughnuts, croissant or other business-type pastries, *churros*, chocolates and candies, nougat and *marzipan*
**Sugar-sweetened beverages (SSB)**	Sugar refreshment, carbonated drinks and packaged fruit drinks and juices

Supplementary Table S2. Baseline anthropometric and biochemical charasterisctic of the participant exluded for microbiota analysis (subjects who consumed between 3 and 5 servings per day of UPFs).

Variable	Subjects who consumed >3 and <5 serv/d of UPFs	Women who consumed >3 and <5 serv/d of UPFs	Men who consumed >3 and <5 serv/d of UPFs
**UPF consumption (serv/ d)**	4.01 ± 0.5	4.0 ± 0.4	3.8 ± 0.4
**Age (y)**	45 ± 1	44 ± 2	45 ± 1
**Smoking**	38	25	13
**Alcohol habit**	109	70	39
**METs**	24.9 ± 2.3	22.9 ± 2.2	30.6 ± 3.4
**Depression prevalence**	5	5	0
**Anxiety prevalence**	7	7	0
**Energy intake (kcal)**	2664 ± 98	2633 ± 88	2752 ± 101
**Energy from UPFs (%)**	15.1 ± 1.7	15.1 ± 1.4	14.9 ± 2.1
**Adherence to MD**	7.5 ± 0.4	7.3 ± 0.5	6.9 ± 0.5
**BMI baseline (kg/m^2)**	29.4 ± 4.8	29.2 ± 4.4	29.9 ± 4.4
**Weight (kg)**	80.9 ± 5.3	77.7 ± 4.8	90.3 ± 5.8
**Waist circumference (cm)**	96 ± 3	93 ± 3	103 ± 5
**Hip circumference (cm)**	108 ± 3	109 ± 4	105 ± 5
**PAS (mmHg)**	124 ± 2	120 ± 2	134 ± 5
**PAD (mmHg)**	77 ± 2	76 ± 3	82 ± 3
**Fat mass (kg)**	28.5 ± 6.3	28.9 ± 6.1	27.3 ± 5.5

**Visceral fat mass (kg)**	1.2 ± 0.4	0.9 ± 0.2	1.9 ± 0.6
**Glucose (mg/dL)**	94 ± 4	92 ± 3	99 ± 4
**Total cholesterol (mg/dL)**	212 ± 4	211 ± 6	213 ± 7
**HDL-cholesterol (mg/dL)**	58 ± 3	61 ± 4	48 ± 2
**LDL-cholesterol (mg/dL)**	68 ± 4	65 ± 4	73 ± 5
**Triglycerides (mg/dL)**	96 ± 6	89 ± 4	117 ± 9
**ALT (U/L)**	23 ± 3	18 ± 3	32 ± 6
**AST (U/L)**	22 ± 1	21 ± 1	25 ± 2
**Insulin (mU/L)**	7.4 ± 2.1	7.4 ± 1.9	7.1 ± 2.2
**Adiponectin (µg/mL)**	11.8 ± 4.2	13.2 ± 3.9	8.2 ± 3.0
**TNF (pg/mL)**	0.8 ± 0.2	0.8 ± 0.1	0.8 ± 0.2
**Leptin (ng/mL)**	31.1. ± 4.7	38.5 ± 5.3	11.8 ± 3.9
**HOMA-IR**	1.8 ± 0.2	1.7 ± 0.2	2.0 ± 0.4
**CRP (µg/mL)**	2.5 ± 0.3	2.6 ± 0.4	2.2 ± 0.2

Variables are expressed as means ± SE for quantitative variables and as numbers of cases for qualitative variables. Differences between groups were assessed by t-test or Mann–Whitney test (METs, glucose, adiponectin, ALT, AST, TNFa, HOMA-IR and CRP) according to the distribution of data and quantitative values variables were assessed by chi squared.
Serv/day: servings per day.
ALT: alanine aminotransferase, AST: aspartate aminotransferase, CRP: C-reactive protein, DBP: diastolic blood pressure, HDL-c: HDL cholesterol,
HOMA-IR: insulin resistance index, LDL-c : LDL cholesterol, MD: Mediterranean diet, MET: metabolic equivalent of tasks, SBP: systolic blood pressure, Serv/d :servings per day, TNF: tumor necrosis factor alpha, TSH: thyroid-stimulating hormone, UPF: ultra-processed food.
Each variable was analyzed using total UPFs consumption adjusted for BMI, age and energy intake, except differences in age, BMI, weight, energy form UPFs and total energy.

Supplementary Table S3. Non-significant bacterial taxa (FDR>0.05) analyzed by EdgeR between subjects who consumed less than 3 and more than 5 servings per day of UPFs.

	log2FC	Pvalues	FDR
	**Genus**		
Turicibacter	-0.9585	0.015324	0.077896
Bacteroides	0.41952	0.018839	0.088398
Holdemania	0.52153	0.044022	0.19181
Paraprevotella	0.7564	0.080406	0.32699
Clostridium	-0.42848	0.088814	0.3386
Dielma	-0.59626	0.10585	0.35948
Alistipes	0.34515	0.10608	0.35948
Bilophila	0.44273	0.11546	0.3707
Barnesiella	-0.53665	0.13994	0.40648
Acidaminococcus	0.75102	0.1673	0.46387
Christensenella	-0.47747	0.20011	0.50986
Oscillospira	-0.36878	0.2006	0.50986
Romboutsia	0.36267	0.22457	0.53338
Phascolarctobacterium	0.54317	0.22734	0.53338
Haemophilus	-0.51229	0.24577	0.55526
Catenibacterium	0.56088	0.2706	0.57915
Dorea	-0.21554	0.2778	0.57915
Blautia	0.22347	0.28483	0.57915
Sutterella	0.51293	0.30339	0.597
Butyrivibrio	-0.46843	0.35303	0.67297
Melainabacter	-0.42192	0.42821	0.77321
Parasutterella	0.30209	0.43337	0.77321
Adlercreutzia	0.25562	0.44702	0.77321
Lachnoclostridium	0.15336	0.45632	0.77321
Howardella	0.22312	0.55574	0.91623
Oscillibacter	-0.095313	0.58016	0.93131
Veillonella	0.20519	0.61143	0.93254
Peptococcus	-0.15082	0.68226	0.93254
Prevotella	-0.18811	0.69092	0.93254
Senegalemassilia	0.12031	0.72999	0.93254
Candidatus_Soleaferrea	-0.15827	0.73814	0.93254
Eubacterium	-0.054164	0.7391	0.93254
Catabacter	-0.11038	0.76723	0.93254
Anaerostipes	-0.14425	0.78042	0.93254
Victivallis	0.1019	0.79862	0.93254
Allisonella	0.094404	0.80763	0.93254

Ruminococcus	0.052355	0.8102	0.93254
Erysipelatoclostridium	-0.082912	0.81323	0.93254
Faecalibacterium	0.039918	0.8163	0.93254
Tyzzerella	0.053546	0.82901	0.93254
Dialister	-0.10781	0.83578	0.93254
Butyricimonas	-0.061085	0.84922	0.93254
Coprococcus	0.028656	0.85056	0.93254
Odoribacter	-0.038453	0.8561	0.93254
Dehalobacterium	-0.041268	0.88126	0.93673
Fusicatenibacter	-0.024237	0.89066	0.93673
Streptococcus	-0.029081	0.93279	0.95033
Eggerthella	-0.024835	0.93476	0.95033
Saccharibacteria	-0.0037541	1	1
	**Family**		
Porphyromonadaceae	0.43077	0.01233	0.098636
Enterobacteriaceae	0.8612	0.056983	0.31802
Bacteroidaceae	0.31836	0.077958	0.35638
Coriobacteriaceae	0.38395	0.092938	0.37175
Rikenellaceae	0.34037	0.10461	0.37195
Desulfovibrionaceae	0.40622	0.13647	0.39261
Mogibacteriaceae	-0.45222	0.13651	0.39261
Clostridiaceae	-0.34612	0.14723	0.39261
Sutterellaceae	0.37967	0.17643	0.39751
Christensenellaceae	0.47014	0.18216	0.39751
Unclassified_Clostridiales	0.50169	0.19243	0.39751
Acidaminococcaceae	0.53209	0.19875	0.39751
Victivallaceae	0.42977	0.28631	0.53894
Pasteurellaceae	-0.43215	0.3151	0.56018
Peptostreptococcaceae	0.26329	0.35898	0.59436
Melainabacteriaceae	-0.47076	0.37147	0.59436
Lachnospiraceae	-0.13184	0.45032	0.6862
Erysipelotrichaceae	-0.16315	0.47712	0.69399
Prevotellaceae	-0.15586	0.70285	0.9309
Catabacteriaceae	-0.1143	0.74462	0.9309
Streptococcaceae	-0.10165	0.74748	0.9309
Peptococcaceae	0.098997	0.77815	0.9309
Oscillospiraceae	-0.043496	0.7869	0.9309
Rs_045	-0.057159	0.83319	0.9309
Veillonellaceae	-0.076243	0.84363	0.9309
Dehalobacteriaceae	-0.039199	0.89564	0.94042
Eubacteriaceae	-0.018218	0.91103	0.94042
Ruminococcaceae	-0.010227	0.94312	0.94312

**Order**			
Desulfovibrionales	0.52781	0.036221	0.19318
Enterobacteriales	0.86802	0.056367	0.22547
Coriobacteriales	0.41669	0.082551	0.24171
Burkholderiales	0.439	0.090643	0.24171
Bacteroidales	0.19821	0.1376	0.31451
Lactobacillales	-0.30047	0.31941	0.61108
RF32	-0.46229	0.34373	0.61108
Selenomonadales	0.18853	0.49201	0.78721
I025	-0.15417	0.55791	0.8115
Melainabacteriales	-0.25078	0.63634	0.83841
Victivallales	0.16264	0.68121	0.83841
Clostridiales	0.040545	0.74484	0.83987
Erysipelotrichales	-0.033183	0.88881	0.88881
**Class**			
Erysipelotrichi	0.67915	0.014214	0.11371
Deltaproteobacteria	0.51205	0.044448	0.23706
Betaproteobacteria	0.43633	0.092721	0.33676
Bacteroidia	0.21163	0.10524	0.33676
Alphaproteobacteria	-0.58778	0.22564	0.54569
Coriobacteriia	0.41763	0.23874	0.54569
Bacilli	-0.28093	0.3547	0.7094
Negativicutes	0.18414	0.49786	0.79143
Melainabacteria	-0.29178	0.58098	0.79143
Gammaproteobacteria	0.16768	0.65982	0.79143
TM7_3	-0.093143	0.71412	0.79143
Erysipelotrichia	-0.081757	0.71512	0.79143
Clostridia	0.040763	0.74002	0.79143
Lentisphaeria	0.13126	0.74197	0.79143
**Phylum**			
Bacteroidetes	0.33027	0.021792	0.087167
Proteobacteria	0.37481	0.039947	0.10653
Firmicutes	0.1405	0.17196	0.27514
Lentisphaerae	0.18315	0.64834	0.86445
TM7	-0.066766	0.86424	0.88413
Melainabacteria	-0.078499	0.88413	0.88413

Log2FC: logarithm 2 fold change (positive value when the abundance increases in group of consumption >5 serv/d of adjusted UPFs); FDR: False Discovery Rate.

Supplementary Table S4. Non-significant bacterial taxa (FDR>0.05) analyzed by EdgeR between women who consumed less than 3 and more than 5 servings per day of UPFs.

	log2FC	Pvalues	FDR
	Genus		
Oxalobacter	1.1599	0.026612	0.14924
Streptococcus	-0.75635	0.028426	0.14924
Veillonella	-0.89863	0.053702	0.24894
cc_115	0.72805	0.057862	0.24894
Saccharibacteria	-0.5763	0.059271	0.24894
Collinsella	0.64797	0.068954	0.27151
Romboutsia	-0.56988	0.088614	0.32839
Turicibacter	-0.75866	0.1167	0.39181
Roseburia	-0.49312	0.1182	0.39181
Bilophila	0.53146	0.12438	0.39181
Christensenella	-0.71317	0.13841	0.41522
Catabacter	-0.59923	0.19195	0.53651
Catenibacterium	0.81834	0.20079	0.53651
Peptococcus	-0.59068	0.20863	0.53651
Victivallis	0.61617	0.21463	0.53651
Blautia	-0.31129	0.22142	0.53651
Eggerthella	-0.5123	0.23518	0.54875
Butyricimonas	-0.4532	0.25347	0.5703
Barnesiella	-0.51035	0.26282	0.57096
Holdemania	0.37302	0.27495	0.57226
Clostridium	-0.34033	0.28159	0.57226
Erysipelatoclostridium	-0.45978	0.29973	0.58028
Granulicatella	0.35219	0.31062	0.58028
Fusicatenibacter	-0.22922	0.31317	0.58028
Prevotella	-0.53648	0.3332	0.59976
Lachnoclostridium	-0.24522	0.34538	0.60441
Eubacterium	-0.19001	0.38745	0.64532
Haemophilus	-0.43256	0.38924	0.64532
Alistipes	0.21102	0.40155	0.64866
Howardella	0.3927	0.41615	0.65544
Faecalibacterium	-0.17142	0.43802	0.67306
Tyzzerella	0.23234	0.49425	0.74138
Oscillospira	-0.22238	0.53015	0.75435
Sutterella	-0.36089	0.54093	0.75435
Dielma	-0.26119	0.55393	0.75435
Ruminococcus	0.15226	0.5652	0.75435

Dorea	-0.13408	0.57474	0.75435
Coprococcus	-0.12733	0.6088	0.78274
Dialister	-0.2692	0.66481	0.83173
Candidatus_Soleaferrea	-0.24139	0.67331	0.83173
Paraprevotella	0.16165	0.75975	0.89631
Parasutterella	0.14444	0.76043	0.89631
Odoribacter	0.080339	0.77325	0.89631
Allisonella	-0.12495	0.78258	0.89631
Rothia	0.085236	0.81035	0.89631
Phascolarctobacterium	0.14786	0.81095	0.89631
Adlercreutzia	-0.070147	0.84896	0.92214
Bacteroides	0.034011	0.86858	0.92746
Oscillibacter	0.016785	0.94016	0.98717
Senegalemassilia	-0.0060053	0.98676	1
Anaerostipes	-0.003831	0.9914	1
Dehalobacterium	-0.0081582	1	1
Family			
Lactobacillaceae	1.5095	0.00091988	0.058901
Melainabacteriaceae	-2.0743	0.0017001	0.058901
Oxalobacteraceae	1.4027	0.0060622	0.05968
Enterobacteriaceae	1.4804	0.0070211	0.05968
Bifidobacteriaceae	0.93625	0.013598	0.092463
Streptococcaceae	-0.79126	0.017924	0.10157
Victivallaceae	0.97609	0.05227	0.25388
Rs_045	-0.56548	0.063949	0.27178
Unclassified_Clostridiales	0.79644	0.10983	0.37554
Porphyromonadaceae	0.34068	0.11045	0.37554
Peptostreptococcaceae	-0.50465	0.12475	0.3856
Mogibacteriaceae	-0.49315	0.22417	0.53536
Prevotellaceae	-0.57496	0.23548	0.53536
Desulfovibrionaceae	0.40042	0.23619	0.53536
Eubacteriaceae	-0.23726	0.25525	0.5424
Veillonellaceae	-0.45428	0.33539	0.62624
Acidaminococcaceae	0.53468	0.34327	0.62624
Erysipelotrichaceae	-0.28425	0.35528	0.62624
Catabacteriaceae	-0.39774	0.36838	0.62624
Carnobacteriaceae	0.26948	0.45145	0.73091
Coriobacteriaceae	0.20949	0.48316	0.7467
Clostridiaceae	-0.1959	0.50902	0.75246
Christensenellaceae	0.22884	0.61102	0.80633
Rikenellaceae	0.11654	0.63187	0.80633
Peptococcaceae	-0.22577	0.63984	0.80633

Bacteroidaceae	-0.097746	0.64412	0.80633
Pasteurellaceae	-0.20829	0.6692	0.80633
Ruminococcaceae	-0.068439	0.68775	0.80633
Lachnospiraceae	-0.071681	0.73806	0.83647
Micrococcaceae	0.081688	0.81698	0.88888
Oscillospiraceae	-0.041361	0.83659	0.88888
Sutterellaceae	-0.055242	0.86409	0.89027
Dehalobacteriaceae	0.018086	0.959	0.959
Order			
Desulfovibrionales	0.7387	0.021049	0.097433
Melainabacteriales	-1.467	0.022926	0.097433
Lactobacillales	-0.61157	0.055204	0.1877
Victivallales	0.83628	0.092873	0.22555
Pasteurellales	-0.78417	0.1066	0.22652
I025	-0.44448	0.12342	0.23312
Burkholderiales	0.34639	0.2379	0.40443
Coriobacteriales	0.30225	0.34814	0.53803
RF32	0.39065	0.517	0.69192
Selenomonadales	0.2215	0.58232	0.69192
Clostridiales	0.089626	0.58953	0.69192
Actinomycetales	0.16548	0.61052	0.69192
Bacteroidales	-0.049846	0.73699	0.78305
Erysipelotrichales	-0.042592	0.89137	0.89137
Class			
Bacilli	-0.89177	0.0068279	0.10149
Melainabacteria	-1.5129	0.018562	0.10149
Actinobacteria	0.73916	0.01903	0.10149
?	-0.49273	0.029949	0.11979
Deltaproteobacteria	0.63639	0.043222	0.13831
TM7_3	-0.56296	0.056301	0.15013
Lentisphaeria	0.68928	0.16416	0.37022
Gammaproteobacteria	0.60013	0.18511	0.37022
Erysipelotrichi	0.47926	0.21793	0.38743
Bacteroidia	-0.11134	0.42999	0.68798
Alphaproteobacteria	0.35687	0.55182	0.73853
Coriobacteriia	0.24621	0.6056	0.73853
Betaproteobacteria	0.14647	0.62249	0.73853
Negativicutes	0.1804	0.64621	0.73853
Erysipelotrichia	-0.084652	0.78153	0.83363
Clostridia	-0.001766	0.99002	0.99002
Phylum			
Proteobacteria	0.49245	0.019688	0.078751

CyanobacteriaMelainabacteria	-1.2146	0.053858	0.1275
Lentisphaerae	0.88548	0.079689	0.1275
TM7	-0.3794	0.27973	0.37298
Firmicutes	0.11117	0.44425	0.50772
Bacteroidetes	0.087331	0.64113	0.64113

Log2FC: logarithm 2 fold change (positive value when the abundance increases in group of consumption >5 serv/d of adjusted UPFs); FDR: False Discovery Rate.

Supplementary Table S5. Non-significant bacterial taxa (FDR>0.05) analyzed by EdgeR between women who consumed less than 3 and more than 5 servings per day of UPFs.

	log2FC	Pvalues	FDR
	Genus		
Bacteroides	1.0775	0.0033593	0.05207
Romboutsia	1.5112	0.0057909	0.071807
Gemmiger	1.3974	0.0093589	0.08904
Clostridium	-1.1993	0.010053	0.08904
Lactobacillus	1.2702	0.013103	0.10154
Lachnoclostridium	0.91369	0.015553	0.10715
Oscillospira	-1.1448	0.029235	0.18125
Turicibacter	-1.5627	0.034899	0.18275
Paraprevotella	1.5486	0.035371	0.18275
Sutterella	1.7487	0.041707	0.18563
Parabacteroides	0.8396	0.043611	0.18563
Adlercreutzia	0.96408	0.047993	0.18563
Eggerthella	1.0868	0.048994	0.18563
Veillonella	1.4308	0.052106	0.18563
Saccharibacteria	0.93266	0.053892	0.18563
Streptococcus	1.1053	0.076648	0.25012
Butyricimonas	0.80558	0.11302	0.32053
Phascolarctobacterium	1.2254	0.11782	0.32053
Melainabacter	1.2892	0.11895	0.32053
Roseburia	-0.67942	0.1233	0.32053
Dielma	-1.085	0.12484	0.32053
Alistipes	0.63498	0.13349	0.32053
Bifidobacterium	0.84769	0.13591	0.32053
Faecalibacterium	0.4955	0.13959	0.32053
Holdemania	0.5913	0.15862	0.34916
Oscillibacter	-0.49912	0.16332	0.34916
Erysipelatoclostridium	0.77414	0.17959	0.37114
Collinsella	0.60807	0.1908	0.37424
Victivallis	-0.91643	0.19664	0.37424

Peptococcus	0.69652	0.19919	0.37424
Butyrivibrio	-1.2023	0.21789	0.39362
Anaerofilum	-0.59333	0.22379	0.39362
Parasutterella	0.80402	0.23408	0.39362
Fusicatenibacter	0.41973	0.2349	0.39362
Allisonella	0.75112	0.25832	0.41868
Catabacter	0.71895	0.26628	0.41868
Coprococcus	0.38566	0.27011	0.41868
Barnesiella	-0.69812	0.28288	0.42776
cc_115	0.45153	0.30856	0.45549
Howardella	-0.55704	0.37711	0.53138
Lachnospira	-0.52758	0.38963	0.53682
Eubacterium	0.25393	0.40694	0.54848
Christensenella	-0.45065	0.42013	0.55422
Odoribacter	-0.32269	0.43706	0.55845
Prevotella	0.57413	0.45387	0.55845
Tyzzerella	-0.31617	0.45715	0.55845
Dorea	-0.32525	0.45937	0.55845
Haemophilus	-0.42298	0.6458	0.77
Ruminococcus	-0.18363	0.7101	0.81193
Oxalobacter	0.20183	0.71997	0.81193
Catenibacterium	0.27376	0.72054	0.81193
Bilophila	-0.19793	0.73552	0.81193
Dehalobacterium	-0.15799	0.74645	0.81193
Acidaminococcus	-0.24665	0.84189	0.8975
Shigella	-0.18959	0.85407	0.8975
Senegalemassilia	-0.097961	0.87489	0.90405
Candidatus_Soleaferrea	-0.13671	0.89148	0.9061
Dialister	-0.015896	0.97884	0.97884
	Family		
Lactobacillaceae	1.1979	0.019354	0.12774
Sutterellaceae	1.0788	0.031387	0.17263
Streptococcaceae	1.1179	0.061828	0.29147
Rs_045	0.85885	0.084824	0.34671
Rikenellaceae	0.67804	0.099021	0.34671
Victivallaceae	-1.1177	0.11676	0.34671
Acidaminococcaceae	1.0686	0.11945	0.34671
Mogibacteriaceae	-0.73076	0.14002	0.34671
Porphyromonadaceae	0.4804	0.14039	0.34671
Peptococcaceae	0.77571	0.14709	0.34671
Bifidobacteriaceae	0.7356	0.20177	0.42339
Melainabacteriaceae	1.0346	0.20528	0.42339

Oscillospiraceae	-0.36413	0.24547	0.4765
Coriobacteriaceae	0.41467	0.32183	0.55897
Eubacteriaceae	0.2455	0.39131	0.61839
Unclassified_Clostridiales	-0.54135	0.39352	0.61839
Veillonellaceae	0.45727	0.44057	0.64482
Catabacteriaceae	0.46558	0.46124	0.64482
Prevotellaceae	0.47354	0.46896	0.64482
Pasteurellaceae	-0.55507	0.52258	0.6898
Dehalobacteriaceae	-0.30958	0.54355	0.68989
Desulfovibrionaceae	-0.29439	0.58003	0.70893
Lachnospiraceae	-0.17727	0.6289	0.7412
Enterobacteriaceae	-0.40133	0.66732	0.75936
Erysipelotrichaceae	-0.12451	0.7537	0.82907
Christensenellaceae	0.13457	0.79082	0.84184
Ruminococcaceae	0.059968	0.82563	0.85143
Oxalobacteraceae	0.034598	0.94168	0.94168
Order			
Bacteroidales	0.72169	0.004629	0.074064
RF32	-2.0138	0.025623	0.20498
Burkholderiales	0.98587	0.042474	0.22653
Pasteurellales	-1.528	0.06967	0.27868
Lactobacillales	0.90133	0.12486	0.29254
Selenomonadales	0.72029	0.12798	0.29254
Bifidobacteriales	0.76186	0.18018	0.36037
Melainabacteriales	0.97117	0.24006	0.40363
Victivallales	-0.80195	0.25227	0.40363
I025	0.49588	0.28013	0.40747
Coriobacteriales	0.2784	0.4627	0.58066
Enterobacteriales	-0.6578	0.47179	0.58066
Desulfovibrionales	-0.29296	0.55177	0.63059
Clostridiales	-0.11624	0.61814	0.65935
Erysipelotrichales	-0.073674	0.8724	0.8724
Class			
Alphaproteobacteria	-2.3022	0.010586	0.084685
Betaproteobacteria	1.0111	0.032476	0.16507
Actinobacteria	0.94971	0.041611	0.16507
Erysipelotrichi	0.63456	0.099874	0.26633
Negativicutes	0.71439	0.12473	0.2851
TM7_3	0.64797	0.15297	0.28683
Bacilli	0.80048	0.16134	0.28683
Lentisphaeria	-0.74606	0.28617	0.45788
Melainabacteria	0.81705	0.31486	0.45798

Gammaproteobacteria	-0.70793	0.36542	0.48723
Deltaproteobacteria	-0.25603	0.62015	0.76326
Coriobacteriia	0.15831	0.75642	0.86448
Erysipelotrichia	-0.047767	0.9211	0.94883
Clostridia	0.012021	0.94883	0.94883
	Phylum		
Actinobacteria	0.89132	0.032458	0.12983
Lentisphaerae	-1.3896	0.051135	0.13636
CyanobacteriaMelainabacteria	1.1834	0.15378	0.30755
TM7	0.57946	0.20678	0.32011
Firmicutes	0.16061	0.24009	0.32011
Proteobacteria	0.32024	0.32597	0.37253

Log2FC: logarithm 2 fold change (positive value when the abundance increases in group of consumption >5 serv/d of adjusted UPFs); FDR: False Discovery Rate.
